# Midecamycin Is Inactivated by Several Different Sugar Moieties at Its Inactivation Site

**DOI:** 10.3390/ijms222312636

**Published:** 2021-11-23

**Authors:** Ru Lin, Li-Li Hong, Zhong-Ke Jiang, Ke-Meng Li, Wei-Qing He, Jian-Qiang Kong

**Affiliations:** 1 NHC Key Laboratory of Biotechnology of Antibiotics, Institute of Medicinal Biotechnology, Chinese Academy of Medical Sciences & Peking Union Medical College, Beijing 100050, China; linru0905@163.com (R.L.); jiangzhongke@126.com (Z.-K.J.); polarismki@163.com (K.-M.L.); 2 Institute of Materia Medica, Chinese Academy of Medical Sciences & Peking Union Medical College (State Key Laboratory of Bioactive Substance and Function of Natural Medicines & NHC Key Laboratory of Biosynthesis of Natural Products), Beijing 100050, China; honglili@imm.ac.cn

**Keywords:** macrolide resistance, midecamycin, glycodiversification, glycosylation inactivation

## Abstract

Glycosylation inactivation is one of the important macrolide resistance mechanisms. The accumulated evidences attributed glycosylation inactivation to a glucosylation modification at the inactivation sites of macrolides. Whether other glycosylation modifications lead to macrolides inactivation is unclear. Herein, we demonstrated that varied glycosylation modifications could cause inactivation of midecamycin, a 16-membered macrolide antibiotic used clinically and agriculturally. Specifically, an actinomycetic glycosyltransferase (GT) OleD was selected for its glycodiversification capacity towards midecamycin. OleD was demonstrated to recognize UDP-D-glucose, UDP-D-xylose, UDP-galactose, UDP-rhamnose and UDP-*N*-acetylglucosamine to yield corresponding midecamycin 2′-*O*-glycosides, most of which displayed low yields. Protein engineering of OleD was thus performed to improve its conversions towards sugar donors. Q327F was the most favorable variant with seven times the conversion enhancement towards UDP-*N*-acetylglucosamine. Likewise, Q327A exhibited 30% conversion enhancement towards UDP-D-xylose. Potent biocatalysts for midecamycin glycosylation were thus obtained through protein engineering. Wild OleD, Q327F and Q327A were used as biocatalysts for scale-up preparation of midecamycin 2′-*O*-glucopyranoside, midecamycin 2′-*O*-GlcNAc and midecamycin 2′-*O*-xylopyranoside. In contrast to midecamycin, these midecamycin 2′-*O*-glycosides displayed no antimicrobial activities. These evidences suggested that besides glucosylation, other glycosylation patterns also could inactivate midecamycin, providing a new inactivation mechanism for midecamycin resistance. Cumulatively, glycosylation inactivation of midecamycin was independent of the type of attached sugar moieties at its inactivation site.

## 1. Introduction

Antibiotics have been applied extensively in the clinic, veterinary medicine and farming as antibacterials. Of these antibiotics, macrolides are an important group of antibiotics, which account for 20% of all antibiotics prescribed. At least 500 kinds of macrolide antibiotics are known, most of which are derived from *Streptomyces* species. The frequently used macrolide antibiotics are erythromycin [1], oleandomycin [2,3], josamycin [4,5], midecamycin [4,6], spiramycin [7,8], roxithromycin [9,10], azithromycin [11,12], and clarithromycin [13,14]. Macrolides exhibit high activity against Gram-positive bacteria, and are thus used widely as excellent bacteriostatic agents due to their low toxicity and broad-spectrum activities. Macrolide antibiotics inhibit protein synthesis by binding to the nascent peptide exit tunnel of the bacterial ribosome, thus leading to growth arrest or cell death. The action mechanism of macrolides is determined by the location of sugars and specific functional groups on their macrolactone rings [15,16,17]. However, as with other antibiotics, the misuse and overuse of macrolides will inevitably result in antibiotic resistance [18], which usually causes the decline or loss of antibiotic efficacy. Antibiotic-resistant infections can be difficult and sometimes impossible to treat, thereby resulting in extended hospital stays, higher medical costs and increased mortality. Antibiotic resistance is regarded as one of the biggest threats to healthcare, veterinary and agriculture industries in our time. Hence, it is urgent to explore the underlying mechanisms of macrolide resistance.

To date, several mechanisms including antibiotics efflux, modifications of the antibiotic targets and antibiotic inactivation have been reported [19,20,21]. Of these mechanisms, antibiotic inactivation is achieved by enzyme-mediated structural modifications, such as phosphorylation, glycosylation and acylation [19,20]. Under the actions of modifying enzymes like phosphotransferases (PTs), glycosyltransferases (GTs) and acyltransferases (ATs), macrolide antibiotics are modified to form derivatives with structural alterations, which can impair target binding [18,19,20]. Enzymatic modification of antibiotics has been known as one of the most common resistance mechanisms, which is extensively present in antibiotic producers [18,22]. Enzymatic modifications are effective strategies to inactivate antibiotics, thereby avoiding their damage to producers, which is also called self-resistance [23]. GT-mediated macrolides inactivation has been observed in diverse producers, like *Streptomyces vendargensis* [24], *S.* *lividans* [25,26,27], *S.* *antibioticus* [28,29,30,31,32], *Saccharopolyspora erythraea* [33], Nocardia Species [34,35], *S.* *hygroscopicus* [36] and *S.ambofaciens* [37]. Many macrolides, such as erythromycin [24,25,27], tylosin [25,27], rosaramicin [25,26,27,28], chalcomycin [26,27], lankamycin [26,27,28], methymycin [26,27,28], pikromycin [26,27], oleandomycin [26,27,28,29], spiramycin [26,27], azithromycin [26,27] and rapamycin [38] could be inactivated by glycosylation modifications. The glycosylation inactivation was deemed as a common self-protection mechanism in these macrolide-producing strains.

Diverse macrolide-inactivating GTs had been isolated and functionally identified. The exemplifying macrolide-inactivating GTs were OleI and OleD from *S. antibioticus* [31], MGT from *S. lividans* [25], BaGT from *Bacillus atrophaeus*, BamGT from *B.* *amyloliquefaciens*, BcGT-1 from *B.cereus*, BgGT from *B.* *glycinifermentans*, BpGT from *B.* *paralicheniformis*, as well as BsGT-1 and BssGT from *B.* *subtilis* [38,39]. These GTs have been demonstrated to be acceptor promiscuous, recognizing multiple macrolides. Both OleI and OleD displayed activity against, for example, oleandomycin, carbomycin, tylosin and erythromycin [31]. MGT could recognize diverse macrolides such as chalcomycin, lankamycin, rosaramicin, methymycin, pikromycin and erythromycin [25,26,27]. Besides acceptor promiscuity, many antibiotic-inactivating GTs like OleI, OleD and MGT showed a wide donor tolerance [40], attaching diverse sugar moieties to an aglycon through a process named glycodiversification.

The underlying mechanisms causing macrolide inactivation was elucidated through the structural identification of glycosylated macrolides. MGT usually inactivates methymycin, erythromycin, azithromycin or tylosin through the glucosylation on 2′-OH in their mycaminosyl moiety [25,26,27]. Likewise, both OleD and OleI inactivate oleandomycin by 2′-glucosylation in the mycaminosyl moiety [28,30,31,32,40,41,42,43]. Nocardia species inactivated chalcomycin and tylosin by the glucosylation at 2′-OH in the mycaminosyl moiety of the two antibiotics [35]. In addition, rapamycin inactivation was proved to be caused by glucosylation at its C-28 or C-40 position [38]. These evidences indicated that glucosylation modification at inactivation sites (C-2′, C-28, C-40) was the main glycosylation inactivation pattern of these macrolides. However, the effect of other glycosylation modifications, such as xylosylation and *O*-GlcNAcylation, on macrolide inactivation is still unknown.

Midecamycin, a naturally occurring 16-membered macrolide, is synthesized from *Streptomyces mycarofaciens* [44,45]. It contains a 16-membered lactone ring, to which a disaccharide moiety 4′-*O*-(α-L-mycarosyl)-β-D-mycaminosyl is attached [46]. Midecamycin is active against both erythromycin-susceptible and efflux-mediated erythromycin-resistant strains [47]. Moreover, midecamycin and its derivatives are also active against mycoplasma species [4,6]. Hence, it has been widely applied in the clinic to treat upper and lower respiratory tract infections [48]. In addition, midecamycin and other macrolides were supplemented in feeds for food-producing animals to treat infectious diseases and promote the health [49,50]. Due to the important roles in clinical therapy and animal-derived food safety, midecamycin resistance has attracted significant attention [34,35]. Midecamycin resistance was due to drug inactivation caused by phosphorylation, reduction, deacylation, or a combination thereof [34,35]. It was not clear, however, that the glycosylation modifications including glucosylation of midecamycin could inactivate midecamycin.

**Figure 1:**
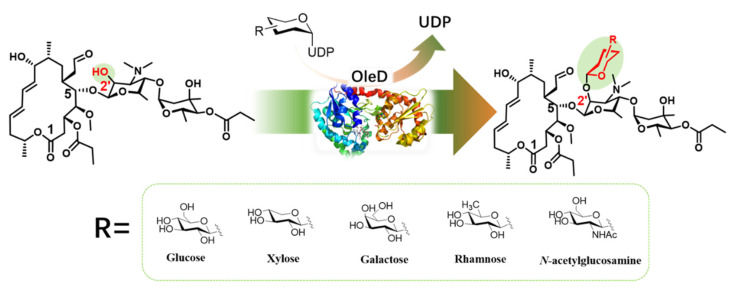
OleD-catalyzed glycodiversification towards midecamycin.

Herein, midecamycin inactivation caused by varied glycosylation modifications was demonstrated. Specifically, OleD was screened from four candidate GTs as the most favorable biocatalyst to glycodiversify midecamycin, generating five 2′-substituted glycosides, most of which had low yields (Figure 1). Protein engineering was then conducted with the aim to improve the conversions of OleD towards five reactive donors. Q327F and Q327A were thus identified as the most favorable biocatalysts capable of increasing the conversion towards UDP-GlcNAc and UDP-Xyl, respectively. OleD, Q327F and Q327A were thus used as the biocatalysts for scale preparation of midecamycin 2′-*O*-glucopyranoside, midecamycin 2′-*O*-acetylglucosamine and midecamycin 2′-*O*-xylopyranoside, respectively. The antimicrobial activities of the three midecamycin 2′-*O*-glycosides were lost completely. These data indicated that glycosylation inactivation could contribute to midecamycin resistance. Besides glucosylation, other glycosylation patterns could result in midecamycin inactivation, suggesting glycosylation inactivation of midecamycin was independent of the type of attached sugar moieties. This study will lay a foundation for the mechanism clarification of macrolide resistance and the development of GT inhibitors as drugs.

## 2. Results

### 2.1. Expression and Purification of GTs

**Figure 2:**
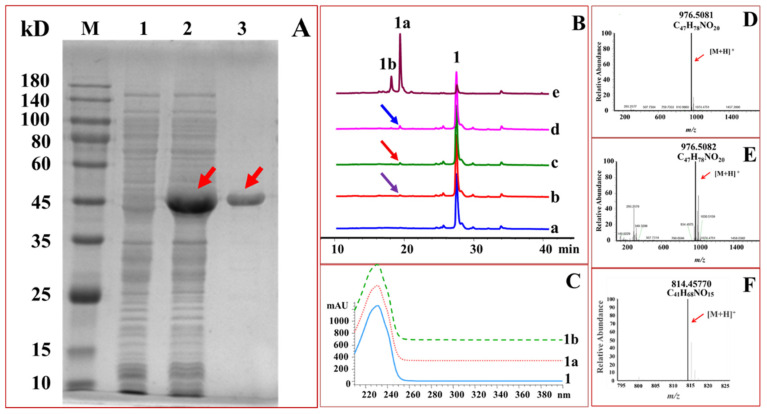
OleD-catalyzed glucosylation towards midecamycin. (**A**), SDS-PAGE analysis of purified OleD. M, Protein marker indicated in kDa in the left margin; 1, Induced *E. coli* harboring pET-His empty vector; 2, Soluble fraction of OleD; 3, The purified OleD. The arrows showed the expressing OleD protein. (**B**), Glucosylation of midecamycin catalyzed by the purified OleD(e), Srm29(d), SpnP(c), DesVII (b) or no protein (a), which were separated by HPLC. 1, 1a and 1b referred to midecamycin and its monoglucosylated products. Arrows showed the glucosylated products. (**C**), UV spectrum of midecamycin (1) and its glucosylated products (1a and 1b). (**D**), HR-MS spectrum of 1a with *m/z* value of 976.5081. (**E**), HR-MS spectrum of 1b with *m/z* value of 976.5082. (**F**), HR-MS spectrum of midecamycin with *m/z* value of 814.4577.

OleD [41], DesVII [51], SpnP [52,53] and Srm29 [54] are macrolide GTs. Hence, these GTs were selected as the candidate biocatalysts for glycosylation of midecamycin. The genes encoding the four GTs were thus induced to express in *Escherichia coli* for preparative production of biocatalysts, respectively. SDS-PAGE analysis showed that an intense band with the expected size of 45.7 kDa was present in the supernatant of the recombinant strain harboring *OleD* gene. On the contrary, no corresponding band was detected in the control supernatant, suggesting *OleD* was successfully expressed in *E. coli* as a soluble product. The soluble OleD protein was subsequently purified to near homogeneity, reaching 107.96 mg/mL (Figure 2A). Likewise, the other three genes *desVII*, *spnP* and *srm29* were demonstrated to be expressed as soluble forms in *E. coli* (Figure S1). The concentration of the purified DesVII, SpnP and Srm29 was 40.32, 50.83 and 46.67 mg/mL, respectively. These recombinant GTs were then used as the biocatalysts to glucosylate midecamycin.

### 2.2. Glucosylation of Midecamycin

Each of these purified OleD, DesVII, SpnP and Srm29 was used as the biocatalyst to react with UDP-Glc and midecamycin, respectively. Results indicated that four GTs could glucosylate midecamycin to form new products (Figure 2B). Of the four GTs, OleD exhibited the most favorable glucosylating activity towards midecamycin. OleD could glucosylate midecamycin to yield two products 1a and 1b (Figure 2B). Conversely, the other three GTs could glucosylate midecamycin to form trace products (Figure 2B). Hence, the purified OleD was used as the biocatalyst for further glycodiversification of midecamycin.

The newly formed peaks 1a and 1b displayed similar UV spectra with that of midecamycin (Figure 2C), suggesting the two products containing similar skeleton structure with that of midecamycin. The newly formed 1a and 1b exhibited [M + H]^+^ ion peaks with *m/z* values of 976.5081 (Figure 2D) and 976.5082 (Figure 2E), respectively, which revealed both were monoglucosylated products of midecamycin with *m/z* value of 814.4577 (Figure 2F). The compound 1a was collected and then analyzed by NMR measurement. ^1^H and ^13^C-NMR data assigned 1a as midecamycin 2′-*O*-glucopyranoside (Table 1, Figures S2–S6), suggesting OleD preferred to attack 2′-OH in mycaminosyl moiety of midecamycin. In addition, OleD was able to glucosylate 2′-OH of oleandomycin [25,26,27,28,38,39,40]. These facts collectively indicated that 2′-OH might be the preferred site of glycosylation for macrolides. In an attempt to collect 1b for structural identification, this compound was found to be unstable and could be easily converted to 1a. It was speculated that there was an isomerization between 1a and 1b, which resulted from the allylic rearrangement caused by two conjugated double bonds in midecamycin. The isomerization of the diene alcohol system in macrolides had been observed previously [55,56]. The instability of 1b, together with its trace amount in the reaction mixture, made it was difficult to purify 1b. Hence, the exact structure of 1b had not been assigned.

The effect of pH and temperature on OleD-catalyzed glucosylation towards midecamycin was illustrated in Figure S7. OleD exhibited a wide pH tolerance ranging from pH 4.0 to pH 12.0. When pH was between 9 and 10, the highest OleD activity was observable. Likewise, OleD had a wide range of temperature tolerance. It kept activity from 0 to 70 °C and got the highest activity at 50 °C. When the temperature was 70 °C, the residual activity of OleD was more than 25%. Hence, the optimal pH 9.0 and optimal temperature 50 °C were determined as the reaction conditions in the following assays unless otherwise specified. The kinetic parameters were determined under the optimal reaction conditions and summarized in Table 2.

### 2.3. OleD-Mediated Glycodiversificaion of Midecamycin

**Figure 3:**
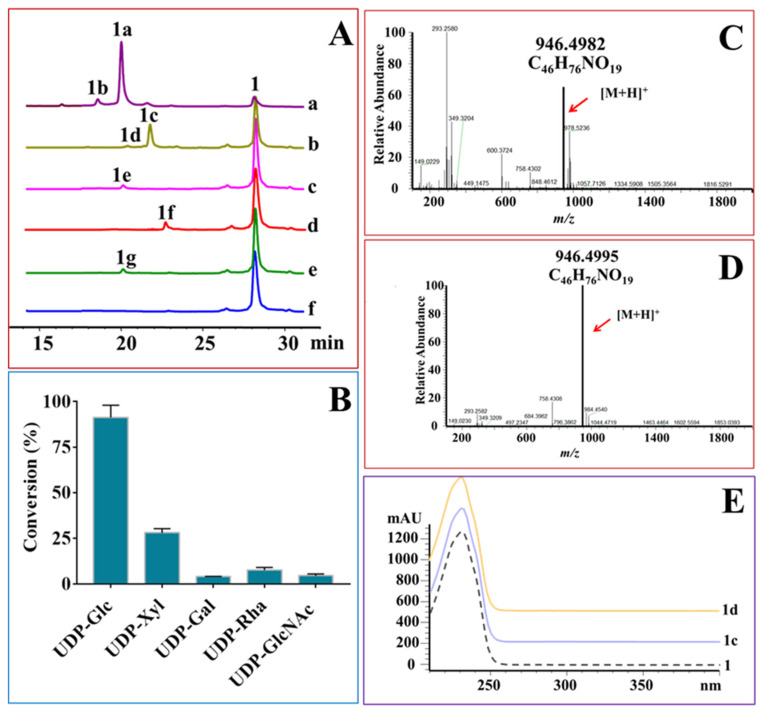
OleD-catalyzed glycodiversification towards midecamycin. (**A**), OleD-catalyzed reaction of midecamycin with UDP-Glc (a), UDP-Xyl (b), UDP-Gal (c), UDP-Rha (d), UDP-GlcNAc (e), or without a donor (f); (**B**), The conversions of midecamycin with UDP-Glc, UDP-Xyl, UDP-Gal, UDP-Rha and UDP-GlcNAc, respectively; (**C**), HR-MS spectrum of 1c with *m/z* value of 946.4982; (**D**), HR-MS spectrum of 1d with *m/z* value of 946.4995; (**E**), UV spectra of 1, 1c and 1d.

The glycodiversification capacity of OleD towards midecamycin was explored with the aim to enzymatically synthesize an array of midecamycin 2′-*O*-glycosides. Each of the compounds summarized in Figure S8 was used as the sugar donor to react with midecamycin under the action of OleD (Figure 3). OleD was reactive with five donors, including UDP-Glc, UDP-Xyl, UDP-Gal, UDP-Rha and UDP-GlcNAc, but stringently repellent to UDP-glucuronic acid (UDP-GlcA), UDP-*N*-acetylgalactosamine (UDP-GalNAc), GDP-mannose (GDP-Man) and other donors in Figure S8. Like UDP-Glc, UDP-Xyl reacted with midecamycin to form two products 1c and 1d displaying similar UV spectra with that of midecamycin (Figure 3A,E). Mass analyses of the two peaks showed *m/z* ions of 946.4995 and 946.4982, respectively, suggesting 1c and 1d were monoxylosylated products of midecamycin (Figure 3C,D). Further NMR analysis assigned 1c as midecamycin 2′-*O*-xylopyranoside (Table 1, Figures S9–S13). The midecamycin xyloside 1d was also unstable, and might be an allylic isomer of 1c (Figure 3A). Moreover, the yield of 1d was very low. Therefore, we did not identify the structure of 1d.

The other three donors, UDP-Gal, UDP-Rha and UDP-GlcNAc, could react with midecamycin to yield one product, respectively (Figure 3A). HRESIMS demonstrated their monoglycosylated products of midecamycin (Figure S14). According to the catalytic behavior of OleD towards midecamycin, these glycosylated midecamycin derivatives 1e, 1f and 1g could reasonably deduced to midecamycin 2′-*O*-galactopyranoside (1e), midecamycin 2′-*O*-rhamnoside (1f) and midecamycin 2′-*O*-acetylglucosamine (1g) (Figure 3). However, more evidences are still needed to determine the exact structure of these monoglycosides. Hence, OleD was demonstrated to glycodiversify midecamycin, attaching five sugar moieties including glucosyl, xylosyl, galactosyl, rhamnosyl and *N*-acetylglucosamine (GlcNAc) at 2′-OH, respectively (Figure 3A). OleD displayed distinct catalytic efficiencies towards these donors (Figure 3B). The conversion of UDP-Glc was the highest, approaching to 91.3%. OleD had low catalytic efficiency for other four donors. The conversion of UDP-Xyl was only 27%, while those of UDP-Gal, UDP-Rha and UDP-GlcNAc were lower, giving 3.96%, 7.62%, 4.56%, respectively (Figure 3B). It was difficult to collect enough amounts of glycosylated products 1c, 1e, 1f and 1g for activity evaluation due to the low conversions. Hence, protein engineering of OleD based on homology modeling was performed with the aim to improve its catalytic efficiency towards UDP-Xyl, UDP-Gal, UDP-Rha or UDP-GlcNAc.

GlcNAc and 2-deoxy-2-fluoroglucose are derivatives of glucose. Moreover, the complex of UGT72B1 (a GT displaying similar structure with that of OleD) and UDP-2-deoxy-2-fluoroglucose (U2F) could provide a reference for homology modeling of OleD with UDP-GlcNAc [57]. Hence, we put the emphasis on the enhancement of the conversion of OleD towards UDP-GlcNAc.

### 2.4. Protein Engineering of OleD to Enhance Its Catalytic Efficiencies

The complex of OleD with U2F was obtained through the alignment and superposition with wild type OleD (PDB ID: 2IYF) and UGT72B1 (PDB ID: 2VCE). Next, the fluoride ion at C-2 of glucose moiety in U2F was replaced with *N*-acetyl group to yield a complex of OleD with UDP-GlcNAc. As observed from the modelled complex structure of OleD with UDP-GlcNAc, the residue Gln331 in OleD formed hydrogen bond with the O3 of GlcNAc, suggesting the critical interaction of a sugar donor with OleD (Figure S15). Gln331 was a component of the strictly conserved signature Glu/Asp-Gln (E/D-Q) and no mutations were therefore performed on this residue [41,57,58]. We focused on amino acids less than 5Å away from Gln331, such as Gln327, Val329, Asp330 and Phe332, which might affect the binding of Gln331 to glycosyl donors (Figure S15). Alanine-scanning mutagenesis of these four residues was thus performed. Four alanine mutants of OleD, namely Q327A, V329A, D330A and F332A, were yielded and their catalytic activities towards UDP-GlcNAc, UDP-Xyl, UDP-Gal, UDP-Rha and UDP-Glc were measured. Q327A variant displayed an improved activity towards UDP-GlcNAc. Conversely, the catalytic activity of the mutant D330A towards the five donors was lost completely. Hence, the residue Q327 was selected for further saturation mutagenesis (Figure S16).

Saturation mutation on Q327 was performed and 19 recombinant OleD mutants were thus induced to express in *E. coli* (Figure S17). Each of these crude mutants was used as the biocatalyst to incubate with midecamycin and UDP-GlcNAc, UDP-Xyl or UDP-Glc, respectively. As illustrated in Figure S18C, the UDP-GlcNAc conversions of 16 mutants increased while the other three mutants, Q327D, Q327E and Q327P, decreased. The most notable increase in UDP-GlcNAc conversion occurred in Q327F mutant. The UDP-GlcNAc conversion of Q327F reached 30.7%, eight times higher than that of the wild OleD (3.6%).

The UDP-Xyl conversion of these variants was also measured (Figure S18B). The conversion of 12 variants towards UDP-Xyl increased, while the other seven obtained a decreased conversion towards UDP-Xyl. The conversion of Q327D and Q327P towards UDP-Xyl decreased significantly. Specifically, the conversion of Q327D declined below 5% while the catalytic activity of Q327P was lost completely. Of 12 mutants with increased activity, Q327I obtained the highest conversion of 46.5% towards UDP-Xyl (Figure S18B), 33% more than that of wild OleD (34.9%).

In addition, the conversion of these variants towards UDP-Glc was also determined (Figure S18A). Of these 19 variants, the conversion of Q327D towards UDP-Glc decreased to 50%, while the catalytic efficiency of Q327P towards UDP-Glc was lost completely. The other 17 mutations at Q327 residue led to an insignificant variation of OleD activity towards UDP-Glc (Figure S18A). Cumulatively, Q327 residue was a favorable site to improve the conversions towards UDP-GlcNAc. The saturation mutations at Q327, however, did not improve the conversion significantly towards UDP-Xyl and UDP-Glc. Thus, the measurement of more residues is required so as to obtain favorable variants with improved conversions towards UDP-Xyl and UDP-Glc. Considering that saturation mutation of Q327 could only significantly increase the conversion towards UDP-GlcNAc, the effects of saturation mutation of Q327 on the conversions towards UDP-Gal and UDP-Rha were thus not tested.

**Figure 4:**
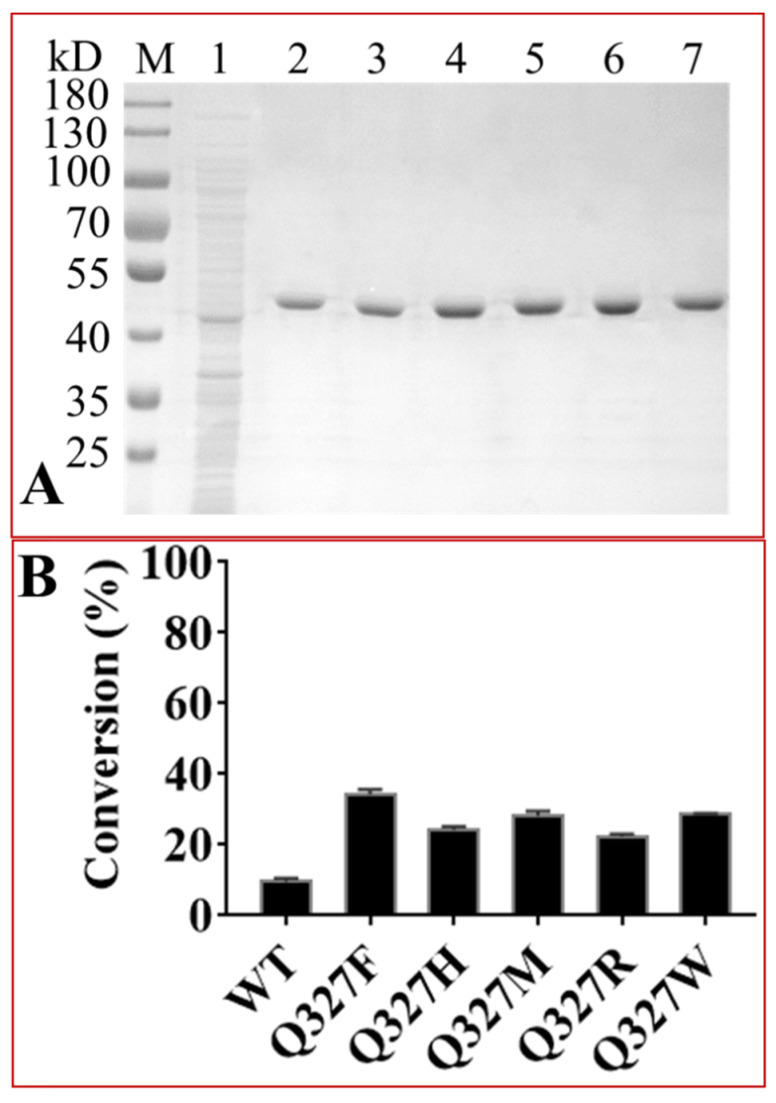
The conversions of OleD and its Q327 mutants towards UDP-GlcNAc. (**A**), SDS-PAGE analyses of the purified OleD and its Q327 variants. M, Protein marker, indicated as kDa in the left margin of the gel; 1, induced *E. coli* harboring pET-His empty vector; 2, wild type OleD, 3, Q327F; 4, Q327H; 5, Q327M; 6, Q327R; 7, Q327W; (**B**), The conversions of Q327 mutants towards UDP-GlcNAc.

In order to further verify these results, five Q327 mutants ranking first to fifth in UDP-GlcNAc conversion were purified (Figure 4A). The concentrations of these purified enzymes were 62.03 (Q327F), 82.52 (Q327H), 99.57 (Q327M), 67.26 (Q327R) and 89.91 mg/mL (Q327W), respectively, and were used as biocatalysts to react with midecamycin and UDP-GlcNAc (Figure 4B). The conversions of these mutants towards UDP-GlcNAc increased significantly. The highest conversion occurred on Q327F variant, reaching 34.13 ± 1.55% (Figure 4).

The wild type OleD and its variants Q327I and Q327F were used as the biocatalysts for scale preparation of midecamycin 2′-*O*-glucopyranoside, midecamycin 2′-*O*-xylopyranoside, and midecamycin 2′-*O*-acetylglucosamine, respectively. These enzymatically synthesized midecamycin 2′-*O*-glycosides were then used to test their water solubility and biological activities.

### 2.5. Water Solubility of Midecamycin 2′-O-Glucopyranoside

Glycosylation modifications usually increase the water solubility of compounds. This notion was further confirmed by the fact that the water solubility of midecamycin 2′-*O*-glucopyranoside was greater than that of midecamycin. The water solubility of the enzymatically synthesized midecamycin 2′-*O*-glucopyranoside was 8.62 mg/mL, 11 times higher than that of midecamycin (0.74 mg/mL).

### 2.6. Antimicrobial Activities of Midecamycin 2′-O-Glycosides

As shown in Table 3, the MIC values of midecamycin against *Bacillus intestinalis* strain T30, *B.* *subtilis* strain 168, *Staphylococcus aureus* and *Streptococcus pneumoniae* were 0.5, 1, 1 and 0.25 μg/mL, respectively, suggesting midecamycin exhibited potent antibacterial activities. Three midecamycin 2′-*O*-glycosides, however, displayed no antibacterial activities against the tested microorganisms listed in Table 3, even if the maximum concentration of these glycosides reached 64 μg/mL. The midecamycin inactivation caused by glycosylation might be due to the configuration change of midecamycin, which in turn prevented the binding of midecamycin to its target [41]. More evidences are required to analyze the exact mechanism underlying the resistance to midecamycin by glycosylation.

## 3. Discussion

Antibiotic resistance is one of the biggest threats to human health and food security. The infections of humans or animals caused by antibiotic-resistant bacteria will increase mortality and medical costs. Understanding more about the resistance mechanisms will be helpful for better treatment of infections caused by antibiotic-resistant bacteria. Glucosylation inactivation is one of the important resistance mechanisms [24,25,26,27,28,29,30,31,32,33,34,35,36,37]. However, if other glycosylation modifications will inactivate antibiotics is not clear. Herein, we took macrolide antibiotic midecamycin as the research object and demonstrated that besides glucosylation, other glycosylation modifications could cause antibiotics inactivation for the first time.

This work has at least three contributions to the new cognition in this field. First, this study provided a novel mechanism for midecamycin inactivation. Midecamycin is an important macrolide antibiotic. The research on its inactivation mechanism contributed to the comprehensive understanding of macrolide resistance. In this manuscript, we convincingly demonstrated glycosylation inactivation was a novel mechanism of midecamycin resistance, which broadened the understanding of midecamycin resistance.

Second, we demonstrated that glycosylation inactivation of midecamycin was independent of the type of attached sugar moieties at its inactivation site. Varied glycosylation modifications, including glucosylation, xylosylation and *O*-GlcNAcylation, could inactivate midecamycin, suggesting varied glycosylation modifications at the same inactivation site could lead to antibiotic resistance.

Finally, *O*-GlcNAcylation improvement of midecamycin was achieved in this investigation. The wild OleD displayed a low *O*-GlcNAcylation efficiency towards midecamycin, which limited the scale preparation of midecamycin 2′-*O*-acetylglucosamine. The protein engineering was performed to enhance the *O*-GlcNAcylation efficiency of OleD. The residue Q327 was identified as the key amino acid regulating *O*-GlcNAcylation of OleD. Q327F was determined as the most favorable OleD variant with seven times conversion enhancement towards UDP-GlcNAc. These data provided a reference for activity improvement of other modifying enzymes.

The facts convincingly demonstrated that varied glycosylation modifications, including glucosylation, xylosylation and *O*-GlcNAcylation could inactivate midecamycin. Besides glycosylation modifications, the resistance to midecamycin could also be achieved by the combinational modification of phosphorylation of 2′-OH and reduction at the 18-formyl group (18-dihydro-2′-*O*-phosphorylmidecamycin) [34,35]. These evidences suggest that multiple modifications at 2′-OH can cause midecamycin inactivation. Hence, 2′-OH is a key determinator for midecamycin activity. 2′-OH of the desosamine moiety, which locates at C5 position of midecamycin and some macrolides, can make specific hydrogen bond interaction with the nucleobase of A2058 of the 23S rRNA, thereby facilitating the occupation of macrolide drugs in the nascent peptide exit tunnel. The glycosylation at 2′-OH might blocked the binding between the desosamine hydroxyl and the N1 atom of A2058, thus leading to macrolide resistance [18,59]. We demonstrated the glycosylation inactivation occurred in midecamycin herein. Still, more evidences are required to determine the exact action mechanism of glycosylation inactivation.

Glycosylation inactivation had been explored comprehensively in *S.* *antibioticus*, the producer of the well-known macrolide oleandomycin [28,29,30,31,43]. An array of GTs capable of glycosylating macrolides had been identified, suggesting macrolide-inactivating GTs might exist extensively in antibiotic-producing organisms [28,29,30,31,43,51,52,53,60]. An OleD isoenzyme might exist in the midecamycin-producing species *S. mycarofaciens* [61]. Further study on this glycosyltransferase will help to understand the inactivation mechanism of midecamycin glycosylation. Cumulatively, this investigation will thus lay a foundation for the mechanism clarification of macrolide resistance and the development of GT inhibitors as drugs.

## 4. Materials and Methods

### 4.1. Chemicals

Midecamycin (1) was purchased from Yuanye Bio-Technology Co., Ltd. (Shanghai, China). The compounds listed in Figure S8 were used as glycosyl donors for glycosylation reactions. These donors were obtained from Sigma-Aldrich Co. LLC (St. Louis, MO, USA), Yuanye Bio-Technology Co., Ltd., J&K Scientific Ltd. (Beijing, China), and Qiyue Biological Technology Co.,Ltd (Xi’an, China), respectively. Acetonitrile and methanol were obtained from MREDA (Beijing, China). Other chemicals and reagents were of analytical grade.

### 4.2. Plasmids and Strains

The *E. coli* strains *Trans*1-T1 and BL21 (DE3) (TransGen Biotech, Beijing, China) were used as the hosts for the plasmid amplification and heterologous expression, respectively. The genes encoding DesVII (accession No. AAC68677.1) [62], OleD (accession No. WP_063854495.1) [40], SpnP (accession NO. AAG23277.1) [63] and Srm29 (accession No. QBG49784.1) [7] were synthesized in Taihe Biotechnology (Beijing, China). The synthetic oleD gene was inserted between *Bam*H Ⅰ and *Eco*R Ⅰ sites of pET-His (Figure S19), while the other three genes were cloned into pColdTF (Takara Bio (Dalian) Co. Ltd., Dalian, China) at the *Xho* Ⅰ and *Hin*d Ⅲ sites to obtain their respective expression vectors (Table S1). The recombinant plasmids were constructed using seamless assembly cloning kit (CloneSmarter Technologies Inc., Houston, TX, USA). The primers used in plasmids construction were listed in Table S2. The authenticity of these recombinant plasmids was verified by direct sequencing.

### 4.3. Protein Expression and Purification

A single colony containing an expression plasmid was grown in 10 mL LB medium supplemented with a final concentration of 0.1 mg/mL ampicillin at 37 °C for 6–8 h. Next, 3 mL cultures were removed and transferred into 300 mL fresh LB medium containing appropriate ampicillin for large-scale cultivation. When the OD_600_ of the cultures reached 0.6–0.8, isopropyl-β-D-thiogalactopyranoside (IPTG) was added with a final concentration of 0.2 mM to induce expression of pETHis-OleD. As for pColdTF-derived plasmids, there was a 30-min rest period at 15 °C prior to 0.2 mM IPTG induction. The cultures continued to grow at 18 °C for additional 20 h after IPTG addition. The cell pellets were collected by centrifugation at 10,625× *g* and resuspended in PBS buffer (20 mM, pH 8.0). The cells were sonicated, and the supernatant was collected by centrifugation at 10,625× *g* and 4 °C for 2 min. The protein expressions were analyzed by the sodium dodecyl sulphate polyacrylamide gel electrophoresis (SDS-PAGE), and purified using Ni-Agarose Resin (CoWin Biotech Co., Ltd., Beijing, China). The concentration of the purified proteins was determined using Nano-300 Micro-Spectrophotometer (Hangzhou Allsheng Instruments Co., Ltd., Zhejiang, China).

### 4.4. Glycosylation Assays

The glycosylation assays were performed in 100 µL PBS buffer (20 mM, pH 8.0) containing 1 mM sugar donor, 1 mM midecamycin (dissolved in DMSO) and 200 μg of a purified GT. After incubated at 37 °C for 2 h, the reactions were terminated by adding the equal volume of methanol and 5 μL glacial acetic acid. The reaction mixtures were separated by centrifugation at 10,625× *g* for 10 min. The resultant supernatants were filtered through 0.22 µm filter and directly monitored by HPLC and HPLC-MS with a C18 column (SilGreen C18, 250 mm × 4.6 mm, 5 μm). The mobile phase consisted of solvent A (10 mM ammonium acetate, pH 8.0) and solvent B (acetonitrile, HPLC grade), with a flow rate of 1.0 mL/min for 35 min. The concentration of solvent B was 10–90% (from 0 to 35 min). The conversion rate (%) was calculated by dividing the peak area of the glycosylation product by the sum of the peak areas of the product and the remaining substrate. The high resolution electrospray ionization mass spectroscopy (HR-ESI-MS) and nuclear magnetic resonance (NMR) data were recorded as described previously [64].

### 4.5. Condition Optimization for OleD-Catalyzed Reactions

The effects of pH and temperature on OleD-catalyzed glucosylation towards midecamycin were explored. The pH dependence of OleD-catalyzed glucosylation was tested in varied buffers including citric acid/sodium citrate buffer (10 mM, pH 3.0–5.6), PBS buffer (20 mM, pH 7.0–8.0) and Na_2_CO_3_-NaHCO_3_ buffer (10 mM, pH 9.0–12.0). All reactions were incubated at 37 °C for 2 h.

The effect of temperature was measured on the optimal optimum Na_2_CO_3_-NaHCO_3_ buffer (10 mM, pH 9.0). Reactions were measured at different temperatures (0, 10, 20, 30, 37, 50, 60, 70 °C) for 2 h.

Kinetic characteristics were tested under the optimal pH and temperature, using different concentrations of midecamycin (0.002–2 mM) and UDP-Glc (0.004–4 mM). Non-linear regression calculations were used to directly determine the apparent kinetic parameters (*K*_m_ and V_max_) by GraphPad Prism 7.0.

### 4.6. Molecular Docking of Ligands with Proteins

Molecular docking of OleD with UDP-GlcNAc was performed using the same procedures as described by Gantt et al. [57].

### 4.7. Directed Mutations of OleD

Using pETHis-OleD plasmid as the template, the directed mutation of OleD was performed in the 2×*TransStart*^®^ *FastPfu* RCR SuperMix according to the direction of the manufacturer (TransGen Biotech.). The primers for OleD mutations were summarized in the supporting Table S2. Cloning mutant products were verified using sanger sequencing. The procedures for protein expression, purification, and glycosylation assays were the same as mentioned above.

### 4.8. Water Solubility Assay

The water solubility of midecamycin and midecamycin 2′-*O*-glucopyranoside was detected using miniaturized shake-flask solubility method [65]. Specifically, midecamycin and midecamycin 2′-*O*-glucopyranoside were added into 100 μL ddH_2_O until turbidity appeared, respectively. After ultrasonic treatment for 30 min, the two mixtures were allowed to stand at room temperature for 3 h. Next, the mixtures were separated by the centrifugation at 10,625× *g* for 30 min, and the resulting supernatants were filtered with 0.22 μM filter membrane. The concentration of filtrate is determined by HPLC.

### 4.9. Evaluation of Antimicrobial Activity of Midecamycin 2′-O-Glycosides

The determination of antimicrobial activity of midecamycin and its glycosides were based on their minimum inhibitory concentrations (MICs) against the tested microorganisms. The MICs of midecamycin 2′-*O*-glycosides were measured using the broth microdilution procedure described previously [66]. Briefly, the cell suspensions of these tested microorganisms were prepared from a 24 h-culture using LB liquid medium (BHI medium for *S. pneumoniae*). The serial dilutions of midecamycin and its glycosides were made using the corresponding mediums in serially decreasing concentrations from 64 μg/mL. The same amount of diluent and bacterial solution (usually 200 μL each) were added to 96 well plate and incubated at 37 °C overnight. The OD_600_ of each well was recorded and the lowest concentration that inhibited bacterial growth completely was defined as MIC values of midecamycin 2′-*O*-glycosides.

## 5. Conclusions

Glucosylation inactivation is one of commonly-accepted resistance mechanisms on antibiotics. Still, the effect of other glycosylation modifications, such as xylosylation and *O*-GlcNAcylation, on macrolide inactivation is unclear. Herein, we demonstrated that besides glucosylation inactivation, other glycosylation could inactivate macrolide antibiotics, deepening the understanding on glycosylation inactivation of antibiotics. In addition, glycosylation modification has been proved to be a new mechanism of midecamycin resistance. Moreover, glycosylation inactivation of midecamycin was independent of the type of attached sugar moieties at its inactivation site. These data contribute to the in-depth understanding of the resistance mechanism and lay a foundation for the development of glycosyltransferase inhibitors.

## Figures and Tables

**Table 1:** NMR data of 1a and 1c in CD_3_OD.

	1a		1c	
Position	δ_C_	δ_H_ (mult, *J* Hz)	δ_C_	δ_H_ (mult, *J* Hz)
1	172.3		172.2	
2	37.9	2.65 (dd, 12.3, 12.0)	37.9	2.65 (overlap)
		2.41 (dd, 13.2, 5.4)		2.37 (dd, 12.3, 7.6)
3	71.1	5.09–5.14 (m)	71.1	5.08–5.13 (m)
4	85.7	3.54 (dd, 10.2, 6.0)	85.9	3.38 (overlap)
5	76.8	3.87 (overlap)	76.3	3.89 (overlap)
6	30.2	2.13–2.21 (m)	30.1	2.14–2.23 (m)
7	31.6	1.48–1.56 (m)	32.6	1.52–1.61 (m)
		1.07–1.13 (m)		0.98–1.03 (m)
8	35.2	1.86–1.95 (m)	35.1	1.92–2.00 (m)
9	74.1	4.23 (dd, 9.6, 5.4)	74.1	4.22 (dd, 9.6, 7.6)
10	128.8	5.64 (dd, 15.6, 9.6)	128.6	5.65 (dd, 15.3, 5.8)
11	136.6	6.58 (dd, 16.0, 10.2)	136.7	6.59 (dd, 15.2, 10.4)
12	133.9	6.10 (dd,15.0, 10.8)	133.8	6.11 (dd, 14.9, 10.6)
13	133.2	5.71–5.78 (m)	133.3	5.71–5.79 (m)
14	41.8	2.13–2.21 (m)	42.8	2.47–2.51 (m)
		2.45–2.50 (m)		2.16–2.22 (m)
15	70.6	4.94 (dd, 13.8, 6.6)	70.7	4.93 (dd, 10.0, 6.5)
16	20.6	1.25 (d, 6.0)	20.9	1.27 (d, 5.6)
17	43.5	2.82–2.90 (m)	43.4	2.83 (dd, 18.6, 11.3)
		2.36 (dd, 18.0, 7.8)		2.36 (overlap)
18	203.5	9.66 (s)	203.5	9.66 (s)
19	15.3	0.99 (t, 5.4)	15.3	0.99 (d, 5.2)
20	175.8		175.8	
21	28.5	2.43–2.49 (m)	28.5	2.41–2.49 (m)
22	9.6	1.19 (overlap)	9.6	1.19 (overlap)
23	62.9	3.62 (s)	62.5	3.58 (s)
1′	102.7	4.69	102.1	4.69 (d, 7.1)
2′	82.3	3.42 (dd, 7.8, 6.0)	81.5	3.48 (dd, 8.9, 5.6)
3′	69.7	2.87 (overlap)	69.9	2.98 (overlap)
4′	80.4	3.63 (overlap)	78.5	3.70 (dd, 9.6, 7.3)
5′	73.5	3.54–3.60 (m)	73.5	3.52–3.56 (m)
6′	19.3	1.34 (d, 6.0)	19.3	1.36 (d, 6.2)
7′ and 8′	41.8	2.59(s)	41.9	2.68 (s)
1″	99.0	5.16 (overlap)	98.7	5.17 (overlap)
2″	43.0	2.06 (d, 15.0)	42.8	2.06 (dd, 14.5, 4.6)
		1.96 (dd, 14.4, 4.8)		1.97 (d, 14.1)
3″	70.4		70.6	
4″	78.6	4.60 (d, 10.2)	78.5	4.62 (d, 9.8)
5″	65.0	4.43–4.47 (m)	65.1	4.37 (overlap)
6″	17.9	1.11 (s)	17.9	1.13 (d, 2.3)
7″	26.8	1.13 (s)	26.5	1.14 (s)
8″	175.8		175.8	
9″	28.3	2.45 (overlap)	28.3	2.46 (overlap)
10″	9.6	1.20	9.6	1.21 (overlap)
1‴	107.8	4.40 (dd, 7.8, 7.2)	107.8	4.88 (d, 7.8)
2‴	76.5	3.21 (dd, 8.4, 8.4)	76.1	3.21 (dd, 9.0, 7.6)
3‴	77.8	3.38 (dd, 9.0, 9.0)	78.0	3.35 (overlap)
4‴	71.3	3.34 (overlap)	71.1	3.46 (overlap)
5‴	78.5	3.29 (overlap)	67.5	3.90 (overlap)
				3.2 (overlap)
6‴	62.9	3.89 (overlap)		
		3.76 (dd, 12.0, 4.8)		

**Table 2:** Kinetic parameters for OleD-catalyzed glucosylation towards midecamycin.

Substrate	*K*_m_ (mM)	V_max_ (mM/min)
UDP-Glc	1.933 ± 0.124	0.080 ± 0.002
midecamycin	0.626 ± 0.106	11.120 ± 0.830

**Table 3:** Antimicrobial activity of midecamycin 2′-*O*-glycosides against microorganisms.

Strain	MIC (μg/mL)			
	Midecamycin	1a	1c	1g
*Bacillus intestinalis* strain T30	0.5	—	—	—
*Bacillus subtilis* strain 168	1	—	—	—
*Staphylococcus aureus*	1	—	—	—
*Streptococcus pneumoniae*	0.25	—	—	—
*Pseudomonas aeruginosa* PAO1	—	—	—	—
*Escherichia coli* DH5α	—	—	—	—

## Data Availability

The datasets analyzed during the current study are available from the corresponding authors on reasonable request.

## Supplementary Materials

The following are available online at https://www.mdpi.com/article/10.3390/ijms222312636/s1.
