# Supplementary material for: Midecamycin Is Inactivated by Several Different Sugar Moieties at Its Inactivation Site
Source: Int J Mol Sci. 2021 Nov 23;22(23):12636. doi: 10.3390/ijms222312636 (PMC8657839; doi:10.3390/ijms222312636)
Supplement: Supplementary file 1 [file ijms-22-12636-s001.zip › ijms-1479819-supplementary.pdf]

SUPPORTING INFORMATION

Midecamycin is inactivated by several different sugar moieties at its inactivation site

Ru Lin[1], Li-Li Hong[2], Zhong-Ke Jiang[1], Ke-Meng Li[1], Wei-Qing He[1,*] , Jian-Qiang Kong[2,*]

1, NHC Key Laboratory of Biotechnology of Antibiotics, Institute of Medicinal Biotechnology, Chinese Academy of Medical Sciences & Peking Union Medical College, Beijing, 100050, China.

2, Institute of Materia Medica, Chinese Academy of Medical Sciences & Peking Union Medical College (State Key Laboratory of Bioactive Substance and Function of Natural Medicines & NHC Key Laboratory of Biosynthesis of Natural Products), Beijing, 100050, China

*, Author to whom correspondence should be addressed.

E-mail: heweiqing@imb.pumc.edu.cn; jianqiangk@imm.ac.cn

Figure legends

Figure S1 SDS-PAGE analysis of the purified SpnP (A), DesVII (B) and Srm29 (C). M, Protein ladder indicated in kDa on the left margin; 1, Soluble fraction containing the target protein; 2, the purified protein. Red arrows indicated the target proteins.

Figure S2 ^{1}H-NMR spectrum (600 MHz) of midecamycin 2'-*O*-glucopyranoside (**1a**)

Figure S3 ^{13}C-NMR spectrum (150 MHz) of midecamycin 2'-*O*-glucopyranoside (**1a**)

Figure S4 COSY spectrum (600 MHz) of midecamycin 2'-*O*-glucopyranoside (**1a**)

Figure S5 HMBC spectrum (600 MHz) of midecamycin 2'-*O*-glucopyranoside (**1a**)

Figure S6 HSQC spectrum (600 MHz) of midecamycin 2'-*O*-glucopyranoside (**1a**)

Figure S7 The effects of pH (A) and temperature (B) on OleD activity

Figure S8 Glycosyl donors used in this study

Figure S9 ^{1}H-NMR spectrum (600 MHz) of midecamycin 2′-*O*-xylopyranoside (**1c**)

Figure S10 ^{13}C-NMR spectrum (150 MHz) of midecamycin 2'-*O*-xylopyranoside (**1c**)

Figure S11 COSY spectrum (600 MHz) of midecamycin 2'-*O*-xylopyranoside (**1c**)

Figure S12 HMBC spectrum (600 MHz) of midecamycin 2'-*O*-xylopyranoside (**1c**)

Figure S13 HSQC spectrum (600 MHz) of midecamycin 2'-*O*-xylopyranoside (**1c**)

Figure S14 The mass spectrum of midecamycin monoglycoside 1e (A), 1f (B) and 1g (C) giving a molecular ion peak at *m/z* 976.5085, 960.5138 and 1017.5350, respectively.

Figure S15 The modelled complex structure of OleD with UDP-GlcNAc. Gln331 and its surrounding residues were positioned in the purple inset.

Figure S16 The effect of alanine-scanning mutagenesis of four residues on the

conversions towards UDP-Glc (A), UDP-Xyl (B), UDP-GlcNAc (C), UDP-Rha (D) and UDP-Gal (E).

Figure S17 SDS-PAGE analyses of OleD and its 19 Q327 variants. Lane M, Protein marker, indicated as kDa in the margin of SDS-PAGE gel; Lane 1-20: OleD, Q327A, Q327C, Q327D, Q327E, Q327F, Q327G, Q327H, Q327I, Q327K, Q327L, Q327M, Q327N, Q327P, Q327R, Q327S, Q327T, Q327V, Q327W, Q327Y.

Figure S18 The effect of 19 Q327 variants on the conversions towards UDP-Glc (A), UDP-Xyl (B) and UDP-GlcNAc(C).

Figure S19 The plasmid map of pET-His

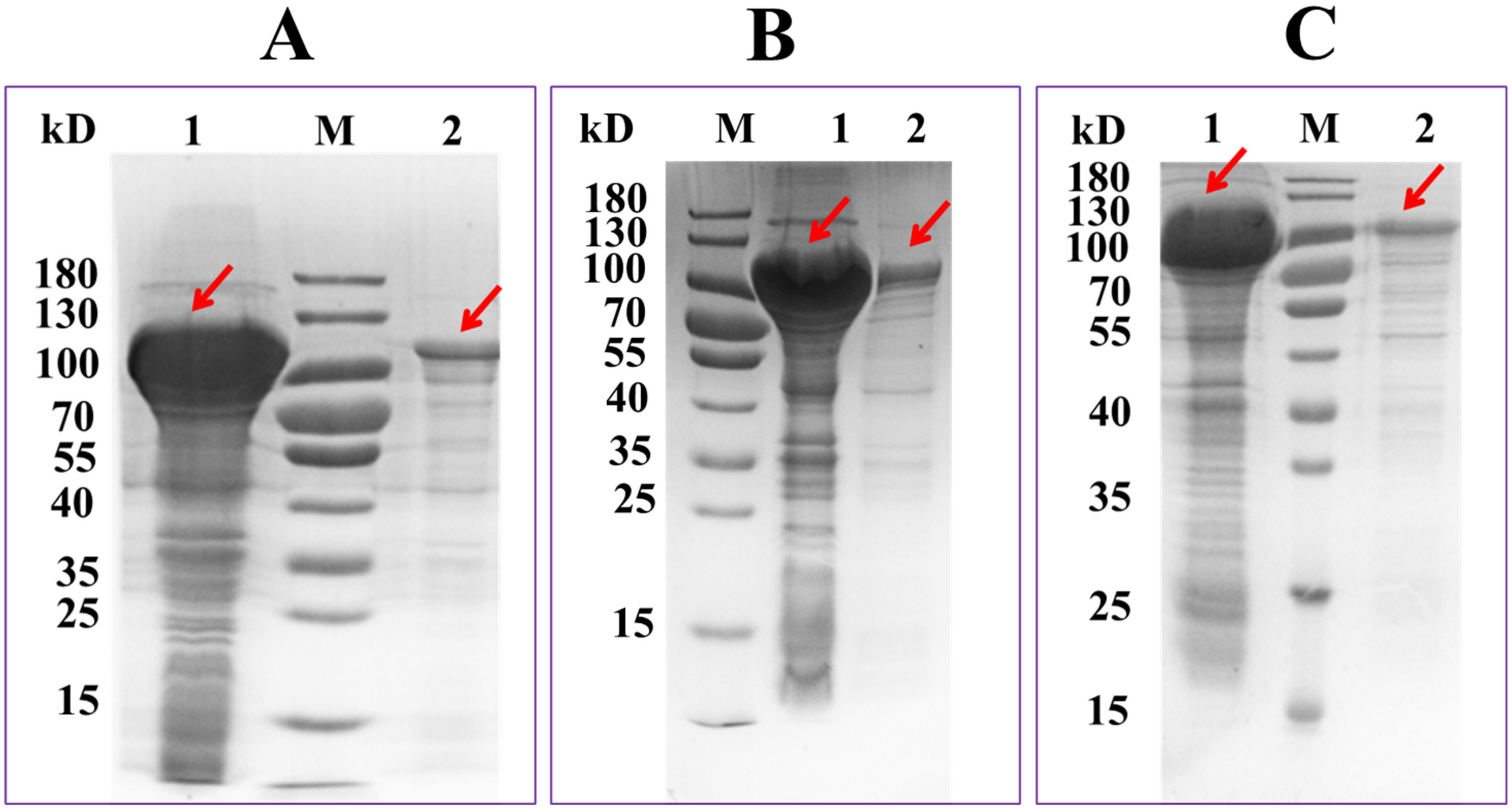

Figure S1 SDS-PAGE analysis of the purified SpnP (A), DesVII (B) and Srm29 (C)

M, Protein ladder indicated in kDa on the left margin; 1, Soluble fraction containing the target protein; 2, the purified protein. Red arrows indicated the target proteins.

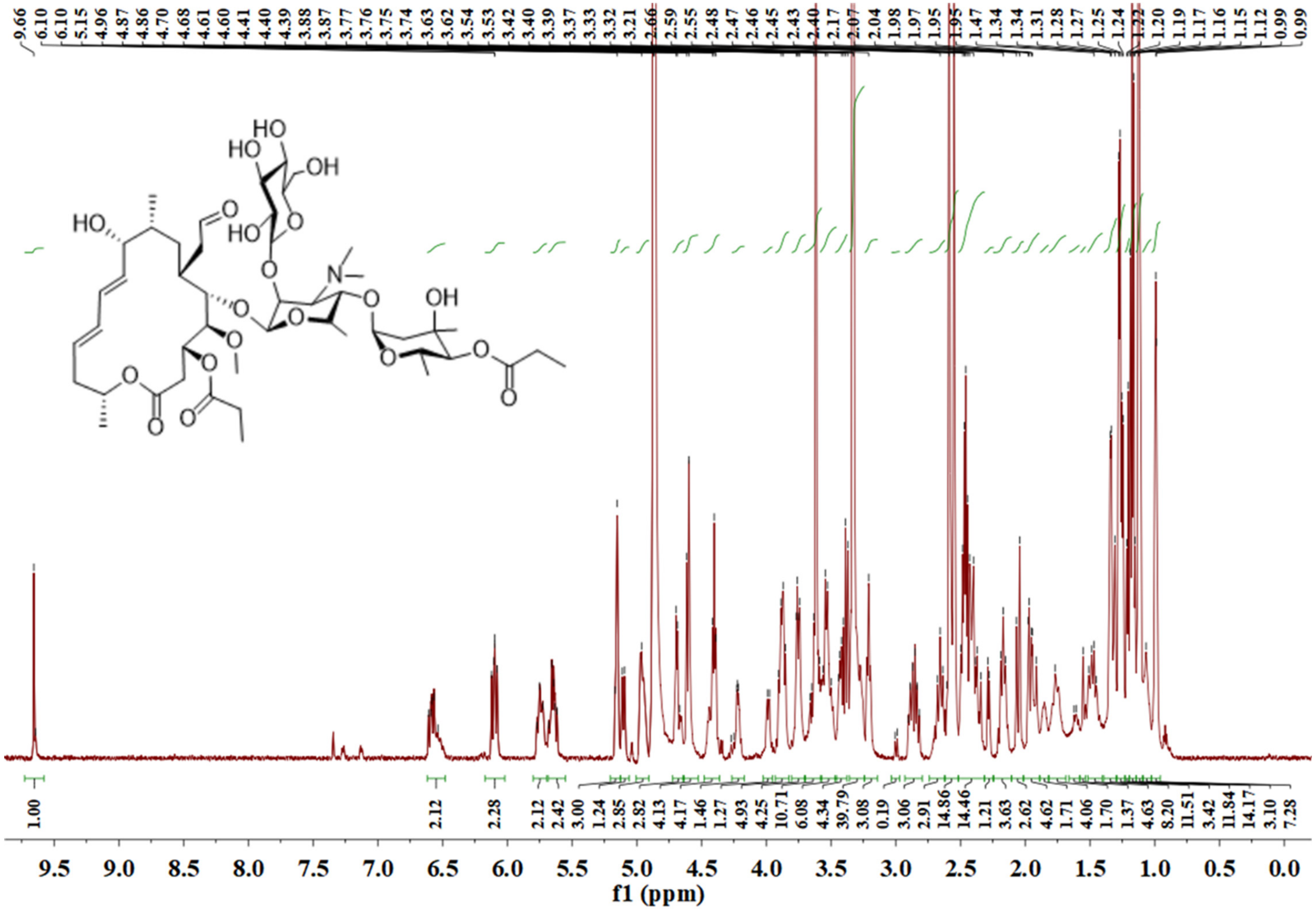

Figure S2 ^{1}H-NMR spectrum (600 MHz) of midecamycin 2'-*O*-glucopyranoside (**1a**)

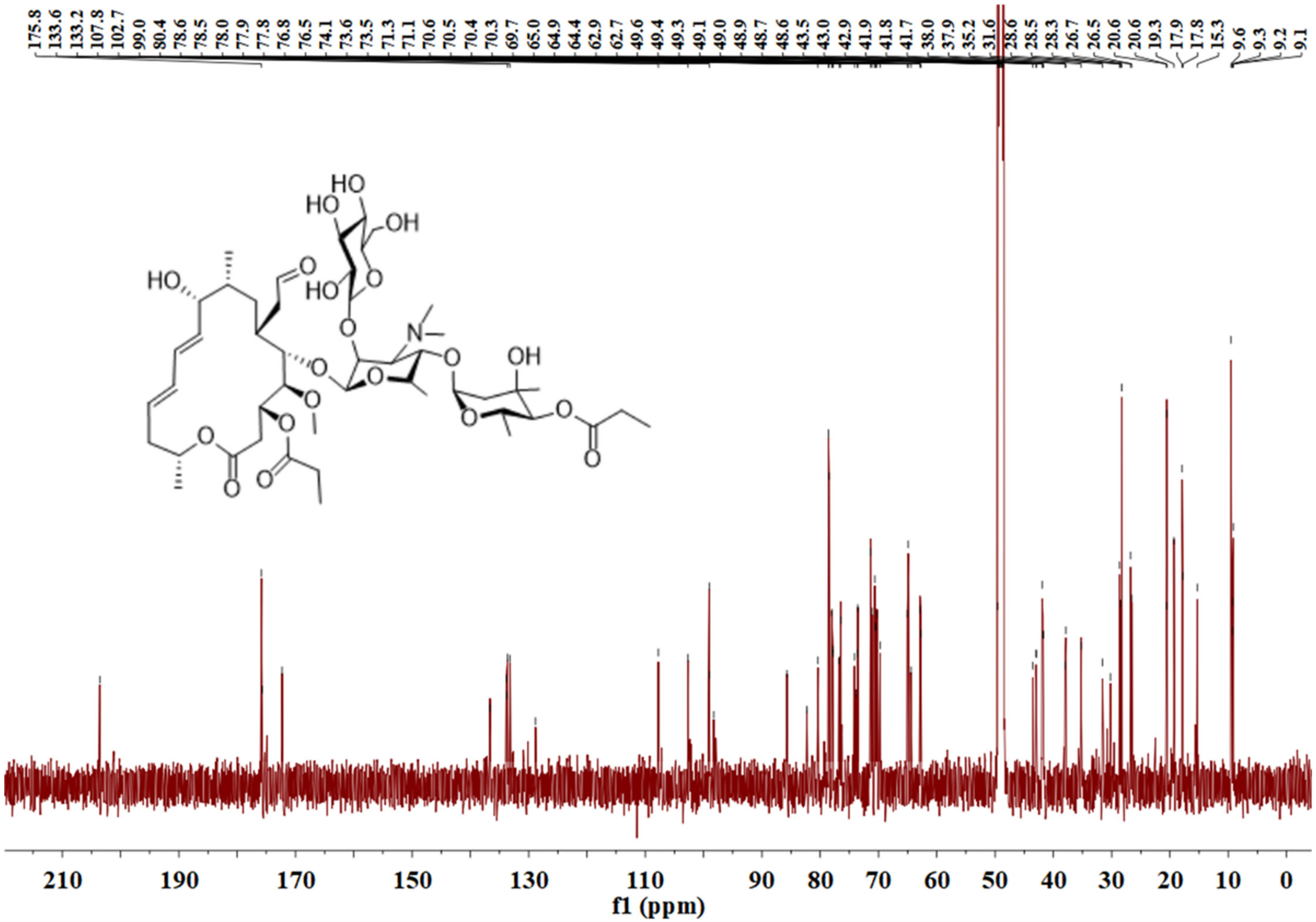

Figure S3 ^{13}C-NMR spectrum (150 MHz) of midecamycin 2'-*O*-glucopyranoside (**1a**)

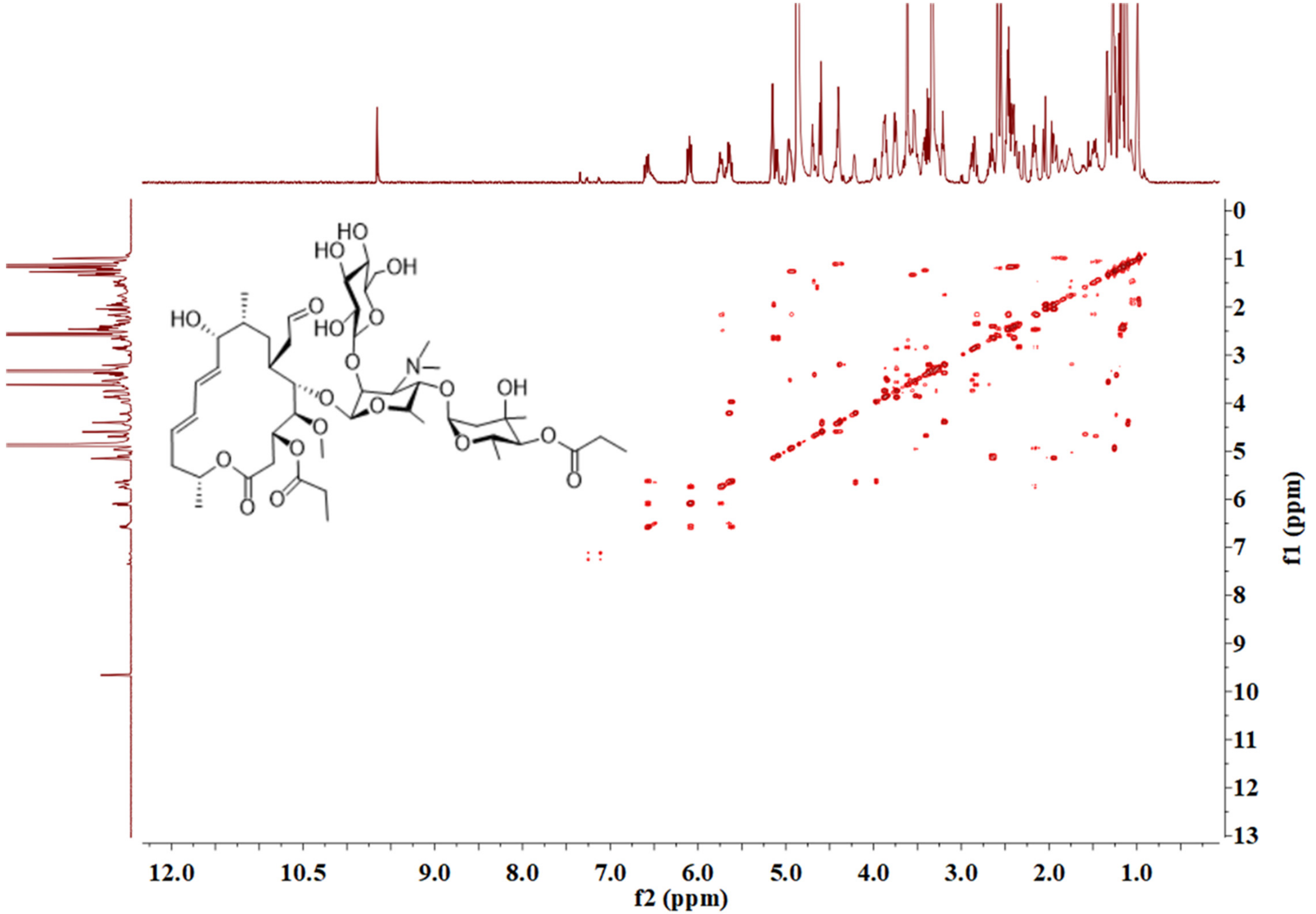

Figure S4 COSY spectrum (600 MHz) of midecamycin 2'-*O*-glucopyranoside (**1a**)

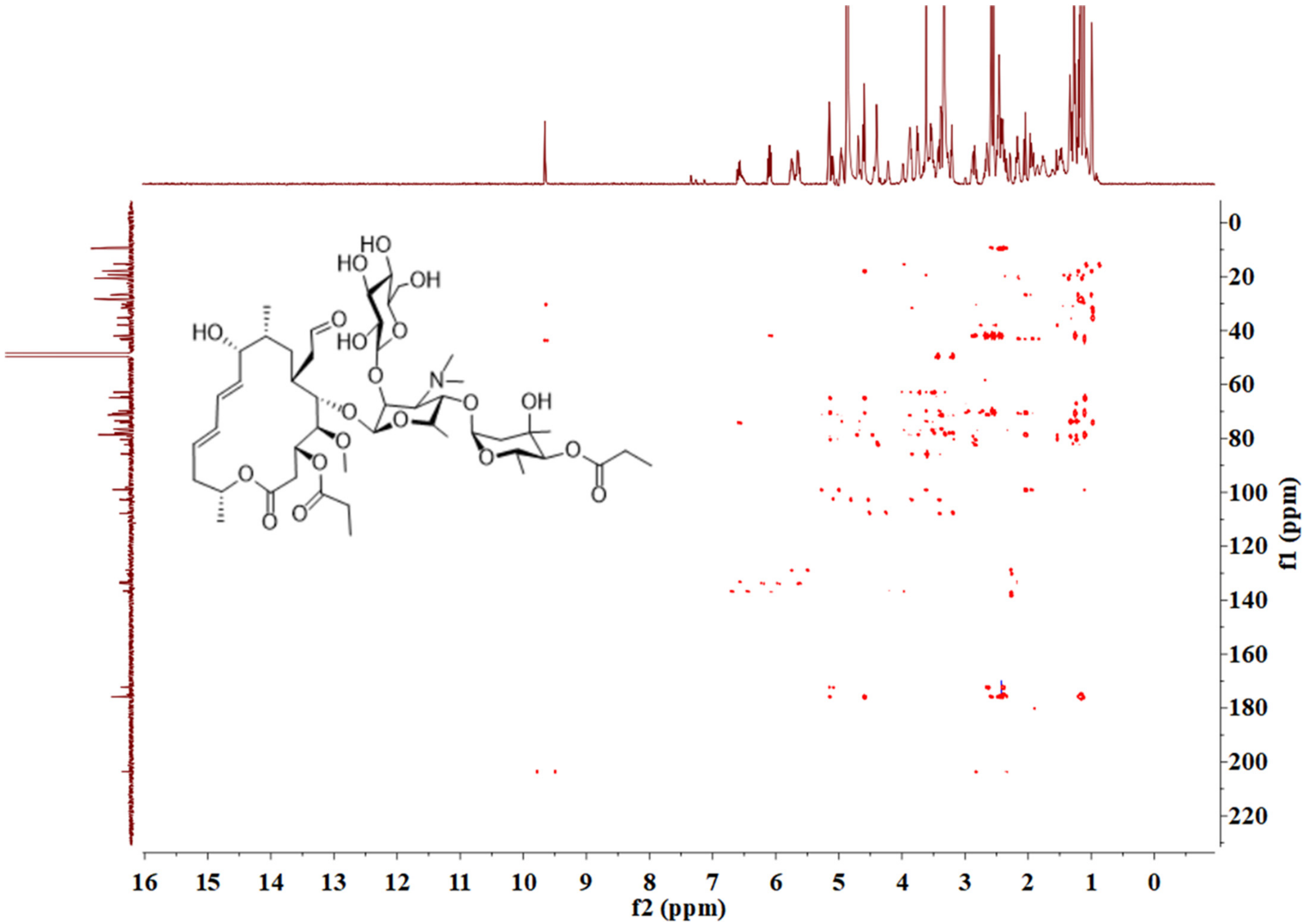

Figure S5 HMBC spectrum (600 MHz) of midecamycin 2'-*O*-glucopyranoside (**1a**)

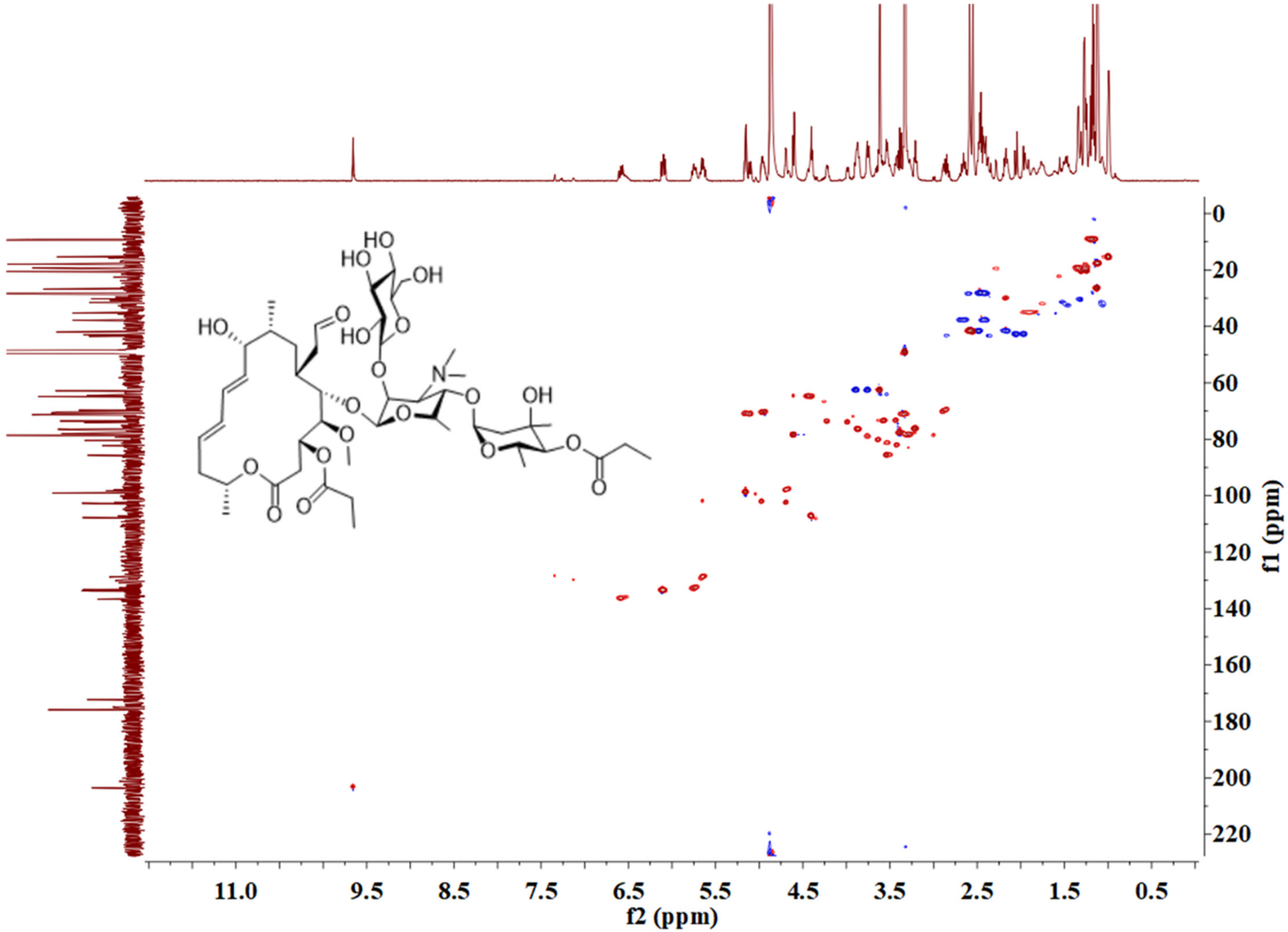

Figure S6 HSQC spectrum (600 MHz) of midecamycin 2'-*O*-glucopyranoside (**1a**)

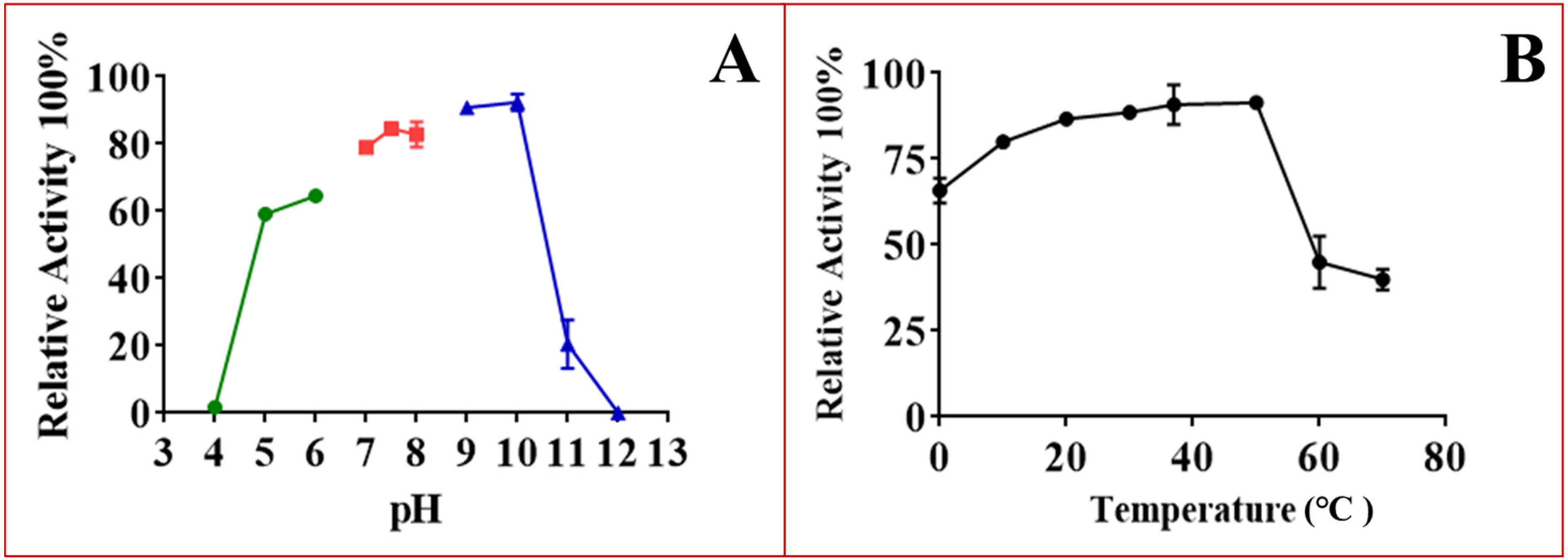

Figure S7 The effects of pH (A) and temperature (B) on OleD activity

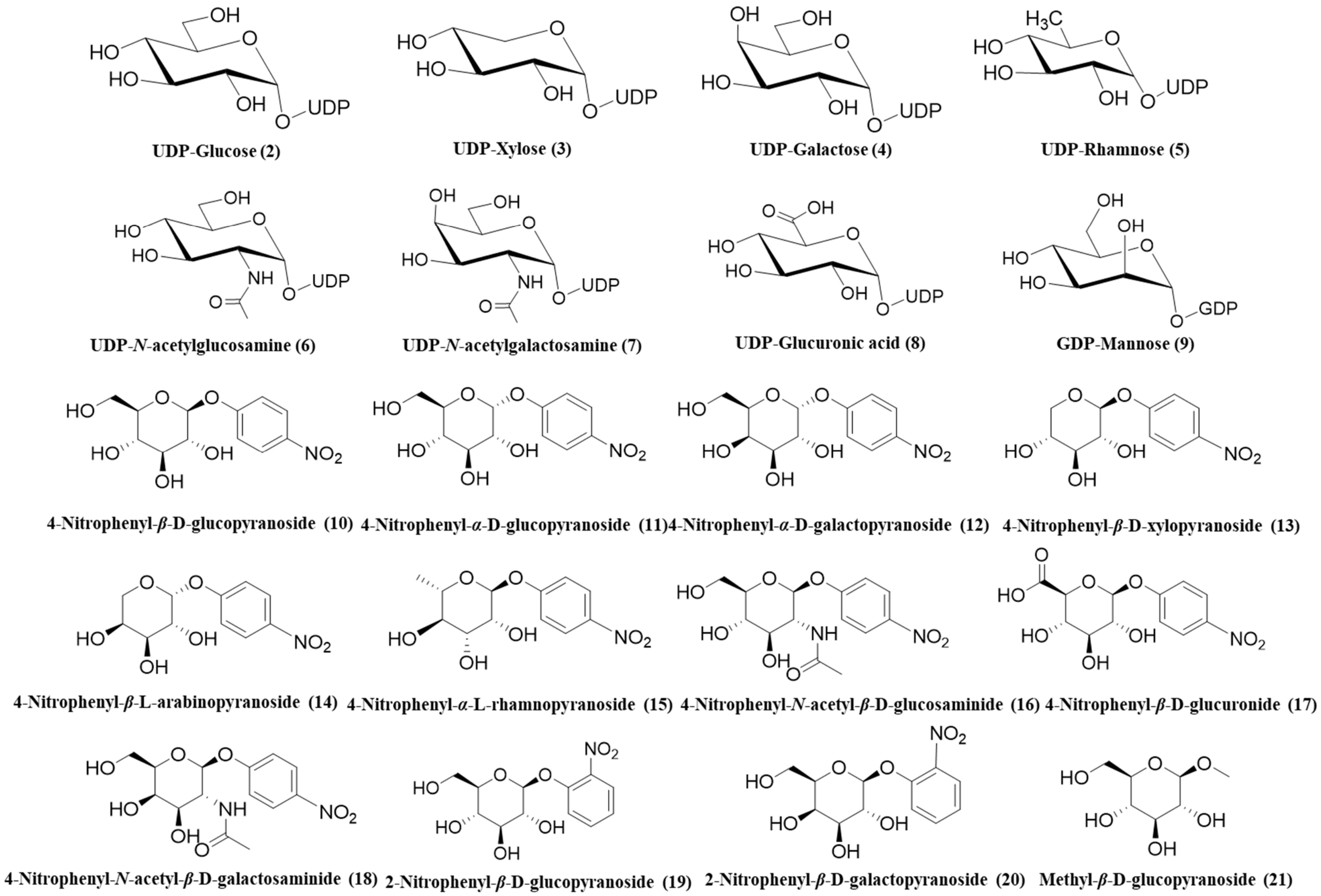

Figure S8 Glycosyl donors used in this study

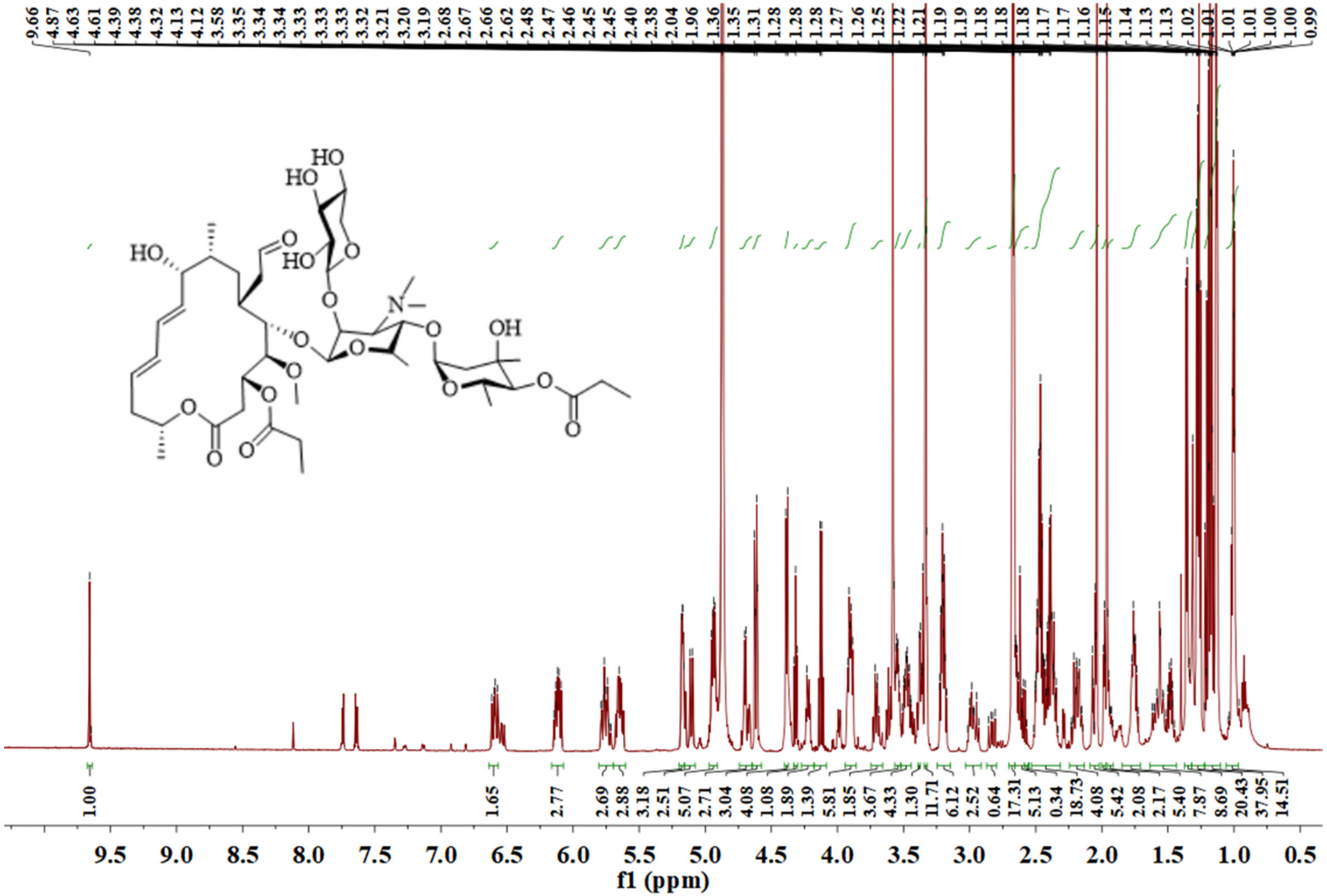

Figure S9 ^{1}H-NMR spectrum (600 MHz) of midecamycin 2'-*O*-xylopyranoside (**1c**)

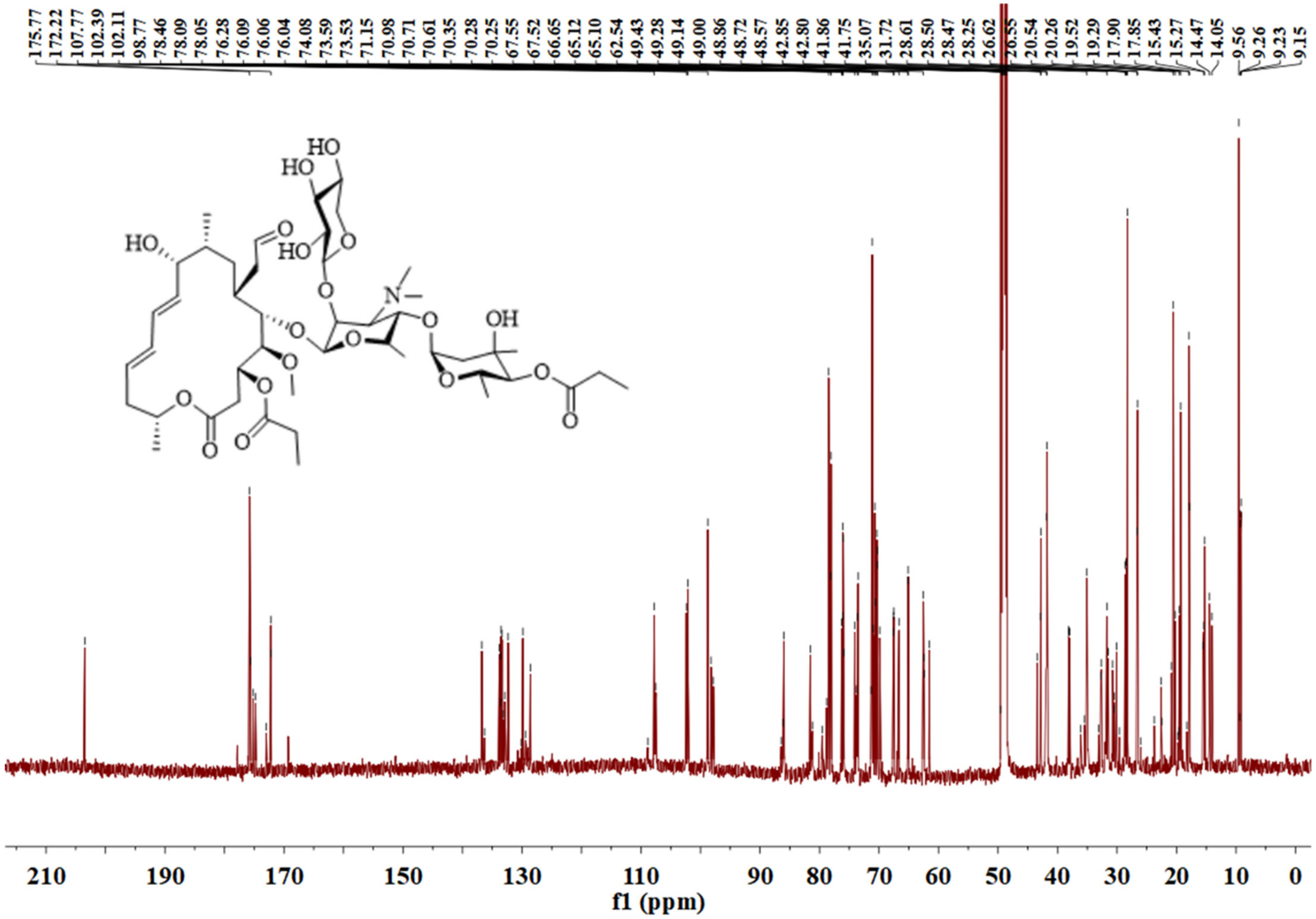

Figure S10 ^{13}C-NMR spectrum (150 MHz) of midecamycin 2'-*O*-xylopyranoside (**1c**)

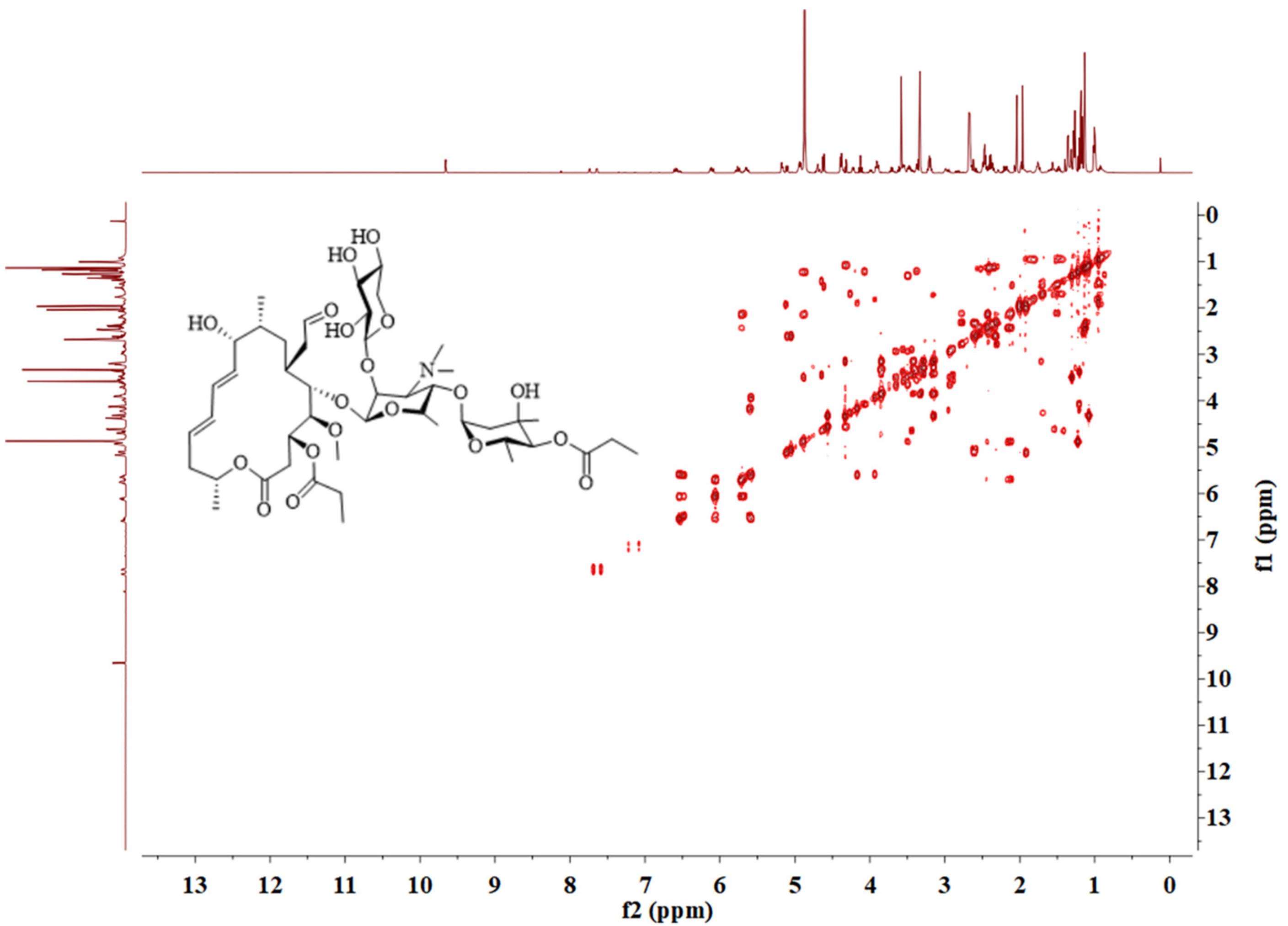

Figure S11 COSY spectrum (600 MHz) of midecamycin 2'-*O*-xylopyranoside (**1c**)

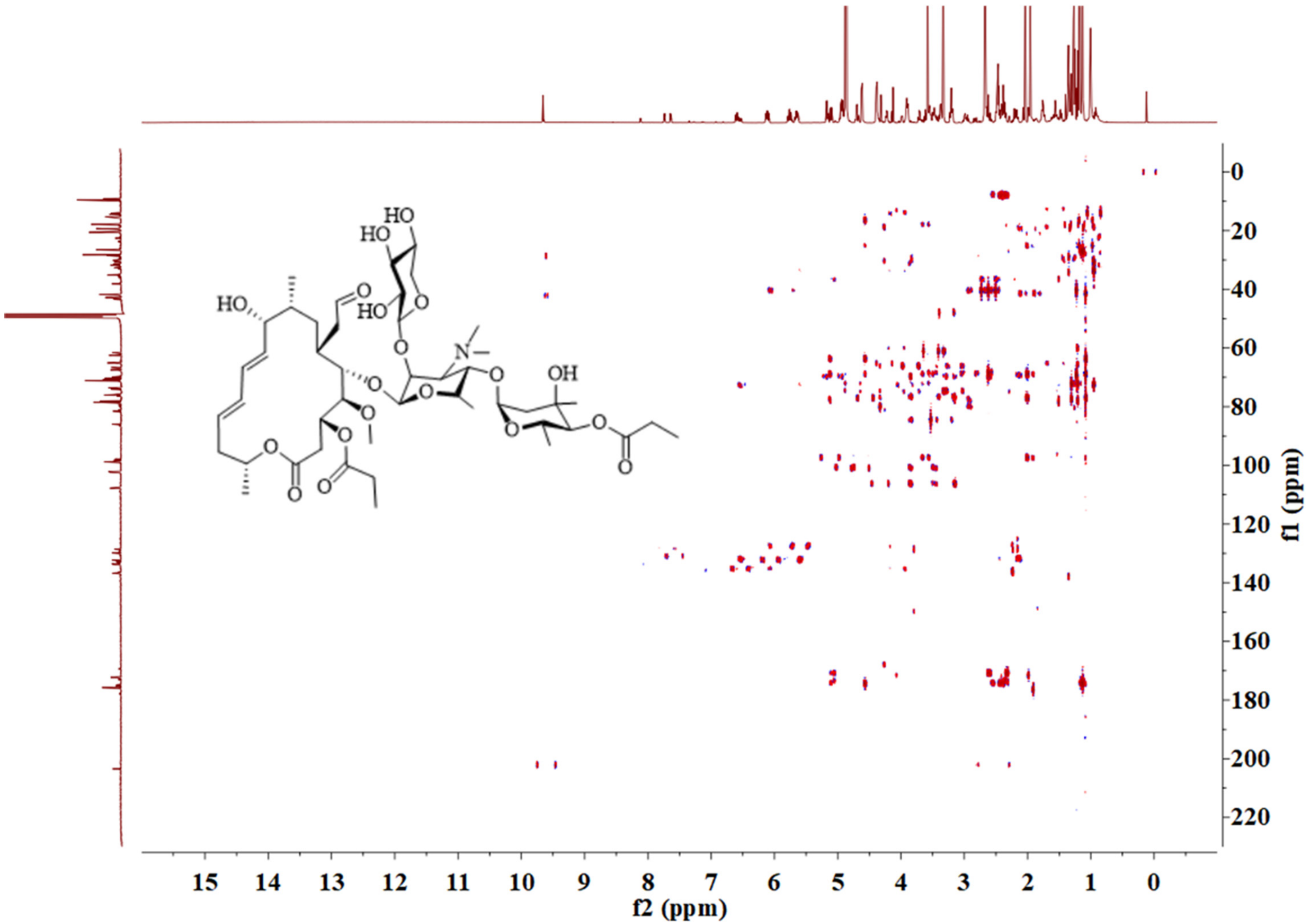

Figure S12 HMBC spectrum (600 MHz) of midecamycin 2'-*O*-xylopyranoside (**1c**)

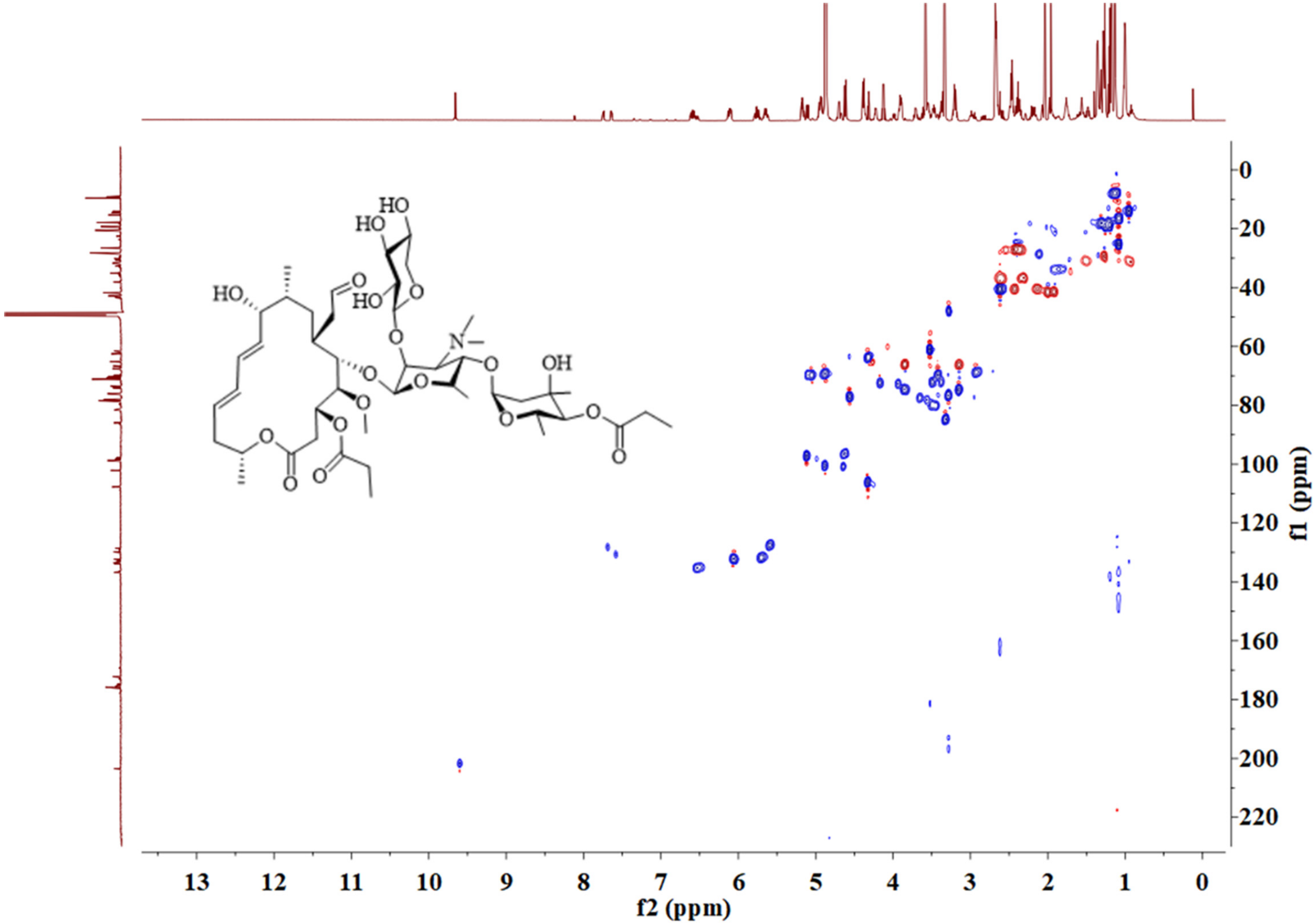

Figure S13 HSQC spectrum (600 MHz) of midecamycin 2'-*O*-xylopyranoside (**1c**)

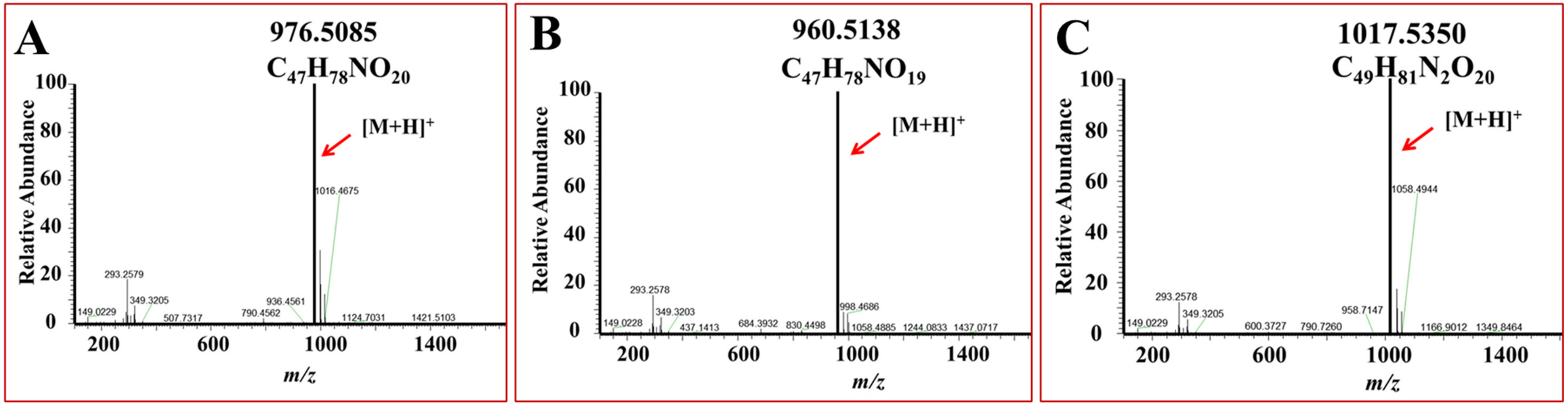

Figure S14 The mass spectrum of midecamycin monoglycosides **1e** (A), **1f** (B) and **1g** (C) giving a molecular ion peak at *m/z* 976.5085, 960.5138 and 1017.5350, respectively.

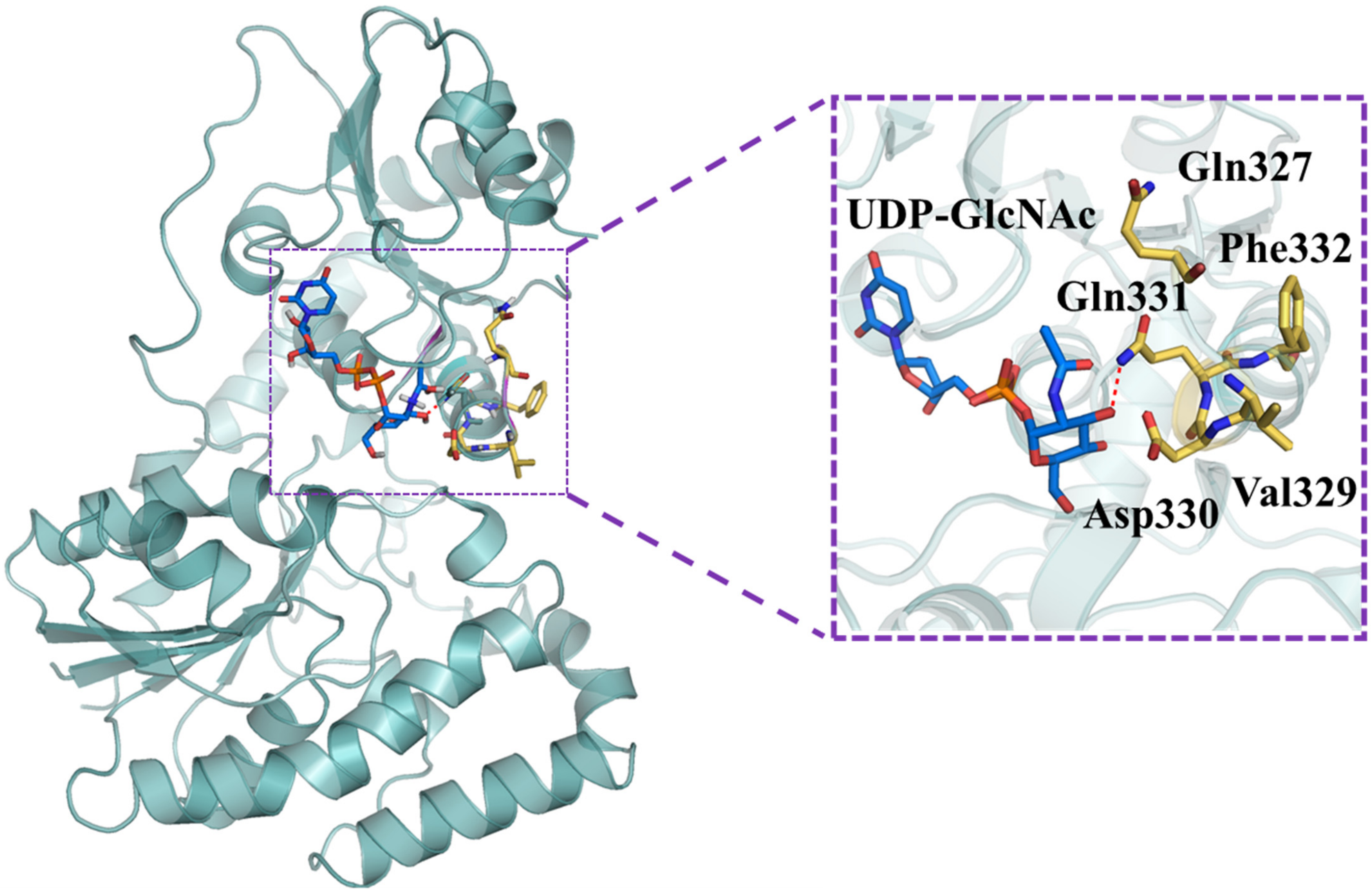

Figure S15 The modelled complex structure of OleD with UDP-GlcNAc. Gln331 and its surrounding residues were positioned in the purple inset

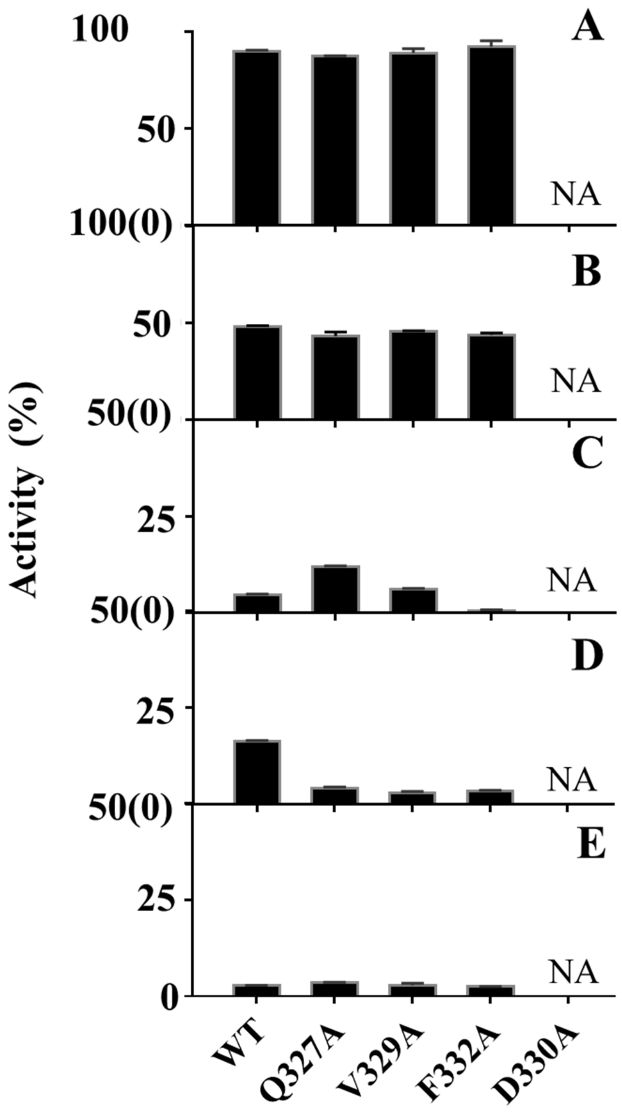

Figure S16 The effect of alanine-scanning mutagenesis of four residues on the conversions towards UDP-Glc (A), UDP-Xyl (B), UDP-GlcNAc (C), UDP-Rha (D) and UDP-Gal (E).

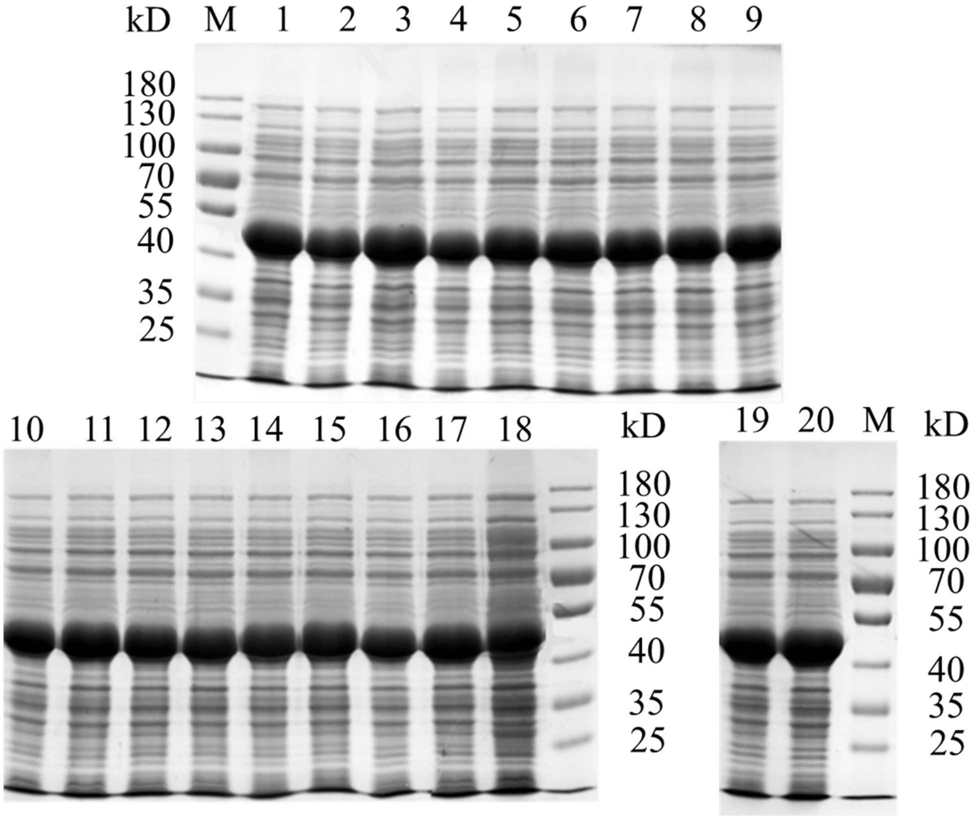

Figure S17 SDS-PAGE analyses of OleD and its 19 Q327 variants
Lane M, Protein marker, indicated as kDa in the margin of SDS-PAGE gel; Lane 1-20: OleD, Q327A, Q327C, Q327D, Q327E, Q327F, Q327G, Q327H, Q327I, Q327K, Q327L, Q327M, Q327N, Q327P, Q327R, Q327S, Q327T, Q327V, Q327W, Q327Y.

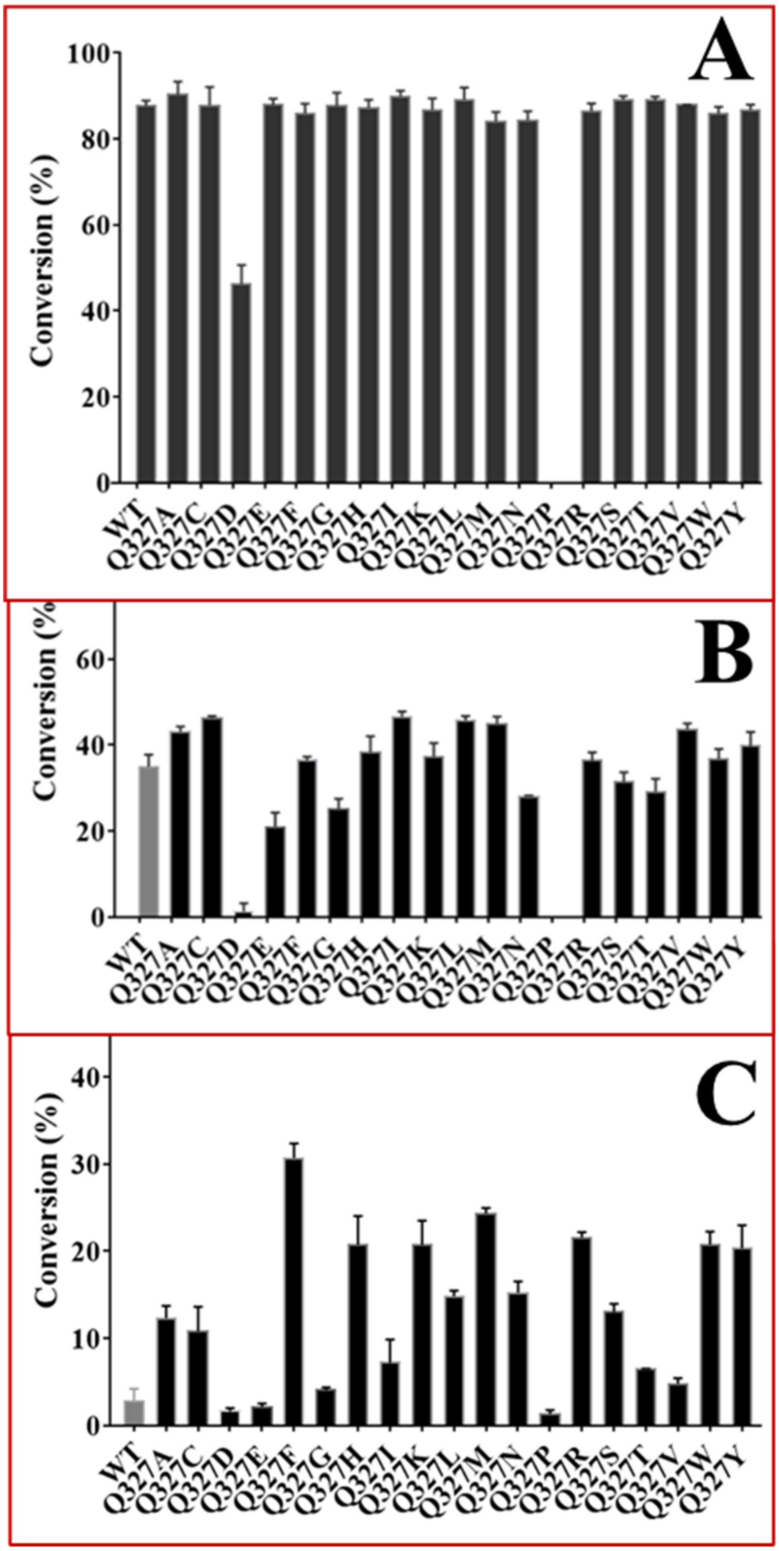

Figure S18 The effect of 19 Q327 variants on the conversions towards UDP-Glc(A), UDP-Xyl (B) and UDP-GlcNAc(C).

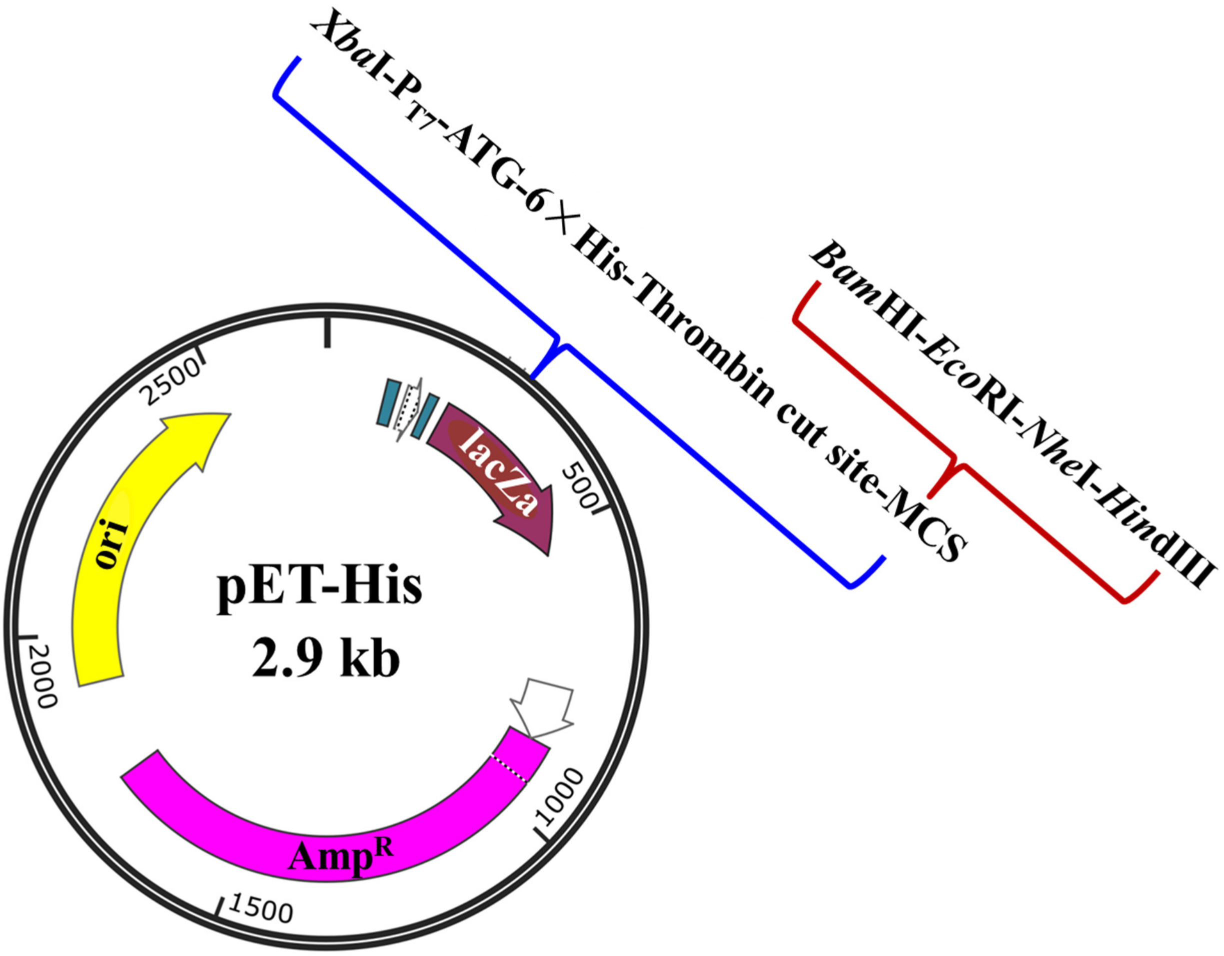

Figure S19 The plasmid map of pET-His

Table captions

Table S1 Plasmids and strains used in this study

Table S2 Primers used in this study

Table S1 Plasmids and strains used in this study

Strains/plasmids	Description	Source/Reference
Strain		
*Trans*1-T1	F^- φ80 (*lac*Z) ΔM15Δ*lac*X74 *hsd*R (r_k^-,m_k^+) Δ*rec*A1398*end*A1*ton*A	TransGen, Beijing, China
BL21(DE3)	F- ompT hsdS(rB-mB-) gal dcm(DE3)	TransGen, Beijing, China
Plasmid		
pET-His	pUC18 derived vector in which an expression cassette containing T7 promoter and MCS was inserted into *Xba*Iand *Hin*dIII sites	This study
pETHis-OleD	pET-His derived vector containing *oleD* gene	This study
pColdTF	A fusion cold shock expression vector that expresses trigger factor (TF) chaperone as a soluble fusion tag	Takara
pColdTF-desVII	pColdTF derived vector containing *desVII* gene	This study
pColdTF-spnP	pColdTF derived vector containing *spnP* gene	This study
pColdTF-srm29	pColdTF derived vector containing *srm29* gene	This study

Table S2 Primers used in this study

Primer	Sequence (5'-3')	Description
*ole*D-F	CGCGGATCCACCACCCAGACCACTCCCG	Forward primer for pETHis-OleD
*ole*D-R	CCGGAATTCTCACCCACCGTTGGGTCGGT	Reverse primer for pETHis-OleD
*srm*29-F	CATATGGAGCTCGGTACCCTCGAGGTCCTCGTGACTTCC	Forward primer for pColdTF-Srm29
*srm*29-R	AGACTGCAGGTCGACAAGCTTTCAGGCACGGCGGTG	Reverse primer for pColdTF-Srm29
*spn*P-F	CATATGGAGCTCGGTACCCTCGAGATGGTGATTCTTGGCATG	Forward primer for pColdTF-Spnp
*spn*P-R	TCTAGACTGCAGGTCGACAAGCTTTCACGGATGGCCATC	Reverse primer for pColdTF-Spnp
*des*VII-F	CATATGGAGCTCGGTACCCTCGAGATGCGCGTCCTGCTGACC	Forward primer for pColdTF-DesvII
*des*VII-R	TCTAGACTGCAGGTCGACAAGCTTTCAGTGCCGGGCGTC	Reverse primer for pColdTF-DesvII
OleD-D330A-F	CCGTACCGCAGGCCGTCGCGCAGTTCGGCAAC	Forward primer for OleD-D330A
OleD-D330A-R	CGCGACGGCCTGCGGTACGGCGATCATGGGCG	Reverse primer for OleD-D330A
OleD-F332A-F	CCGCAGGCCGTCGACCAGGCGGGCAACGCCGAC	Forward primer for OleD-F332A
OleD-F332A-R	CGCCTGGTCGACGGCCTGCGGTACGGCGATCAT	Reverse primer for OleD-F332A
OleD-V329A-F	ATCGCCGTACCGCAGGCCGCGGACCAGTTCGG	Forward primer for OleD-V329A
OleD-V329A-R	CGCGGCCTGCGGTACGGCGATCATGGGCGTCG	Reverse primer for OleD-V329A
OleD-Q327A-F	CCCATGATCGCCGTACCGGCGGCCGTCGACCAGT	Forward primer for OleD-Q327A
OleD-Q327A-R	GCCGGTACGGCGATCATGGGCGTCGCGGTGGC	Reverse primer for OleD-Q327A
OleD-Q327H-F	CCCATGATCGCCGTACCGCATGCCGTCGACCAGT	Forward primer for OleD-Q327H
OleD-Q327H-R	ATGCGGTACGGCGATCATGGGCGTCGCGGTG	Reverse primer for OleD-Q327H
OleD-Q327R-F	CCCATGATCGCCGTACCGCGCGCCGTCGACCAGT	Forward primer for OleD-Q327R
OleD-Q327R-R	GCGCGGTACGGCGATCATGGGCGTCGCGGTG	Reverse primer for OleD-Q327R
OleD-Q327K-F	CCCATGATCGCCGTACCGAAAGCCGTCGACCAGT	Forward primer for OleD-Q327K
OleD-Q327K-R	TTTCGGTACGGCGATCATGGGCGTCGCGGTGG	Reverse primer for OleD-Q327K
OleD-Q327E-F	CCCATGATCGCCGTACCGGAAGCCGTCGACCAGT	Forward primer for OleD-Q327E
OleD-Q327E-R	TTCCGGTACGGCGATCATGGGCGTCGCGGTGGC	Reverse primer for OleD-Q327E
OleD-Q327D-F	CCCATGATCGCCGTACCGGATGCCGTCGACCAGT	Forward primer for OleD-Q327D
OleD-Q327D-R	ATCCGGTACGGCGATCATGGGCGTCGCGGTGGC	Reverse primer for OleD-Q327D
OleD-Q327N-F	CCCATGATCGCCGTACCGAACGCCGTCGACCAGT	Forward primer for OleD-Q327N
OleD-Q327N-R	GTTCGGTACGGCGATCATGGGCGTCGCGGTGGC	Reverse primer for OleD-Q327N
OleD-Q327M-F	CCCATGATCGCCGTACCGATGGCCGTCGACCAGT	Forward primer for OleD-Q327M
OleD-Q327M-R	ATCGGTACGGCGATCATGGGCGTCGCGGTGGC	Reverse primer for OleD-Q327M
OleD-Q327C-F	CCCATGATCGCCGTACCGTGCGCCGTCGACCAGT	Forward primer for OleD-Q327C
OleD-Q327C-R	GCACGGTACGGCGATCATGGGCGTCGCGGTGGC	Reverse primer for OleD-Q327C
OleD-Q327T-F	CCCATGATCGCCGTACCGACCGCCGTCGACCAGT	Forward primer for OleD-Q327T
OleD-Q327T-R	GGTCGGTACGGCGATCATGGGCGTCGCGGTGG	Reverse primer for OleD-Q327T
OleD-Q327S-F	CCCATGATCGCCGTACCGAGCGCCGTCGACCAGT	Forward primer for OleD-Q327S
OleD-Q327S-R	GCTCGGTACGGCGATCATGGGCGTCGCGGTGG	Reverse primer for OleD-Q327S
OleD-Q327W-F	CCCATGATCGCCGTACCGTGGGCCGTCGACCAGT	Forward primer for OleD-Q327W
OleD-Q327W-R	CACGGTACGGCGATCATGGGCGTCGCGGTGG	Reverse primer for OleD-Q327W
OleD-Q327Y-F	CCCATGATCGCCGTACCGTATGCCGTCGACCAGT	Forward primer for OleD-Q327Y
OleD-Q327Y-R	ATACGGTACGGCGATCATGGGCGTCGCGGTGGC	Reverse primer for OleD-Q327Y
OleD-Q327F-F	CCCATGATCGCCGTACCGTTTGCCGTCGACCAGT	Forward primer for OleD-Q327F
OleD-Q327F-R	AAACGGTACGGCGATCATGGGCGTCGCGGTGGC	Reverse primer for OleD-Q327F
OleD-Q327P-F	CCCATGATCGCCGTACCGCCGGCCGTCGACCAGT	Forward primer for OleD-Q327P
OleD-Q327P-R	GGCGGTACGGCGATCATGGGCGTCGCGGTGG	Reverse primer for OleD-Q327P
OleD-Q327I-F	CCCATGATCGCCGTACCGATTGCCGTCGACCAGT	Forward primer for OleD-Q327I
OleD-Q327I-R	AATCGGTACGGCGATCATGGGCGTCGCGGTGG	Reverse primer for OleD-Q327I
OleD-Q327L-F	CCCATGATCGCCGTACCGCTGGCCGTCGACCAGT	Forward primer for OleD-Q327L
OleD-Q327L-R	AGCGGTACGGCGATCATGGGCGTCGCGGTGG	Reverse primer for OleD-Q327L
OleD-Q327V-F	CCCATGATCGCCGTACCGGTGGCCGTCGACCAGT	Forward primer for OleD-Q327V
OleD-Q327V-R	ACCGGTACGGCGATCATGGGCGTCGCGGTGGC	Reverse primer for OleD-Q327V
OleD-Q327G-F	CCCATGATCGCCGTACCGGGCGCCGTCGACCAGT	Forward primer for OleD-Q327G
OleD-Q327G-R	GCCCGGTACGGCGATCATGGGCGTCGCGGTGGC	Reverse primer for OleD-Q327G
